# Innate Immune Cells: A Potential and Promising Cell Population for Treating Osteosarcoma

**DOI:** 10.3389/fimmu.2019.01114

**Published:** 2019-05-16

**Authors:** Zenan Wang, Zhan Wang, Binghao Li, Shengdong Wang, Tao Chen, Zhaoming Ye

**Affiliations:** ^1^Department of Orthopedics, Musculoskeletal Tumor Center, The Second Affiliated Hospital, Zhejiang University School of Medicine, Hangzhou, China; ^2^Institute of Orthopedic Research, Zhejiang University, Hangzhou, China

**Keywords:** osteosarcoma, innate immune cell, adoptive cell therapy (ACT), vaccine, immmune checkpoint

## Abstract

Advanced, recurrent, or metastasized osteosarcomas remain challenging to cure or even alleviate. Therefore, the development of novel therapeutic strategies is urgently needed. Cancer immunotherapy has greatly improved in recent years, with options including adoptive cellular therapy, vaccination, and checkpoint inhibitors. As such, immunotherapy is becoming a potential strategy for the treatment of osteosarcoma. Innate immunocytes, the first line of defense in the immune system and the bridge to adaptive immunity, are one of the vital effector cell subpopulations in cancer immunotherapy. Innate immune cell-based therapy has shown potent antitumor activity against hematologic malignancies and some solid tumors, including osteosarcoma. Importantly, some immune checkpoints are expressed on both innate and adaptive immune cells, modulating their functions in tumor immunity. Therefore, blocking or activating immune checkpoint-mediated downstream signaling pathways can improve the therapeutic effects of innate immune cell-based therapy. In this review, we summarize the current status and future prospects of innate immune cell-based therapy for the treatment of osteosarcoma, with a focus on the potential synergistic effects of combination therapy involving innate immunotherapy and immune checkpoint inhibitors/oncolytic viruses.

## Introduction

Osteosarcoma is the most common primary malignant bone tumor and it often leads to pulmonary metastasis, which is the major cause of death of osteosarcoma patients (1). Surgical resection combined with neoadjuvant and postoperative chemotherapy has increased long-term survival rates to 70% for patients with localized osteosarcomas, but < 20% for patients with recurrent and/or metastasized osteosarcomas. The current standard treatment strategy has remained unchanged for decades (2). Therefore, there is urgent need to develop novel therapies to improve the overall survival rates of osteosarcoma patients, particularly those experiencing relapse and/or metastasis.

Immunotherapy is becoming an attractive therapeutic strategy for the treatment of osteosarcoma. The human immune system, which consists of innate and adaptive immunity, plays a critical role in suppressing tumor growth. The major effector cells in adaptive immunity targeting osteosarcoma are cytotoxic T cells (CTLs). A previous study demonstrated that CTLs played an important role in immune surveillance in osteosarcoma patients (3). In addition, adoptive transfer of T cells successfully resulted in tumor inhibition in mouse models of osteosarcoma (4–6).

Recently, the role of innate immune cells in the control of tumor progression has been characterized. Innate immune cells contribute to tumor suppression through direct recognition and killing, through self-activation to trigger a strong adaptive immune response, or through both mechanisms (7). The antitumor immunocompetence of innate immune cells provides a rational basis for innate immune cell-based therapy, which has shown promise for the treatment of hematopoietic malignancies and solid tumors (8). Indeed, successful treatment of osteosarcomas in preclinical studies using innate immune cells has been reported (9, 10). Our previous studies have shown that innate immune cells were effective against osteosarcoma (11–14). In this paper, we describe the anti-osteosarcoma roles of the following major classes of innate immune cells: dendritic cells (DCs), macrophages, natural killer (NK) cells, natural killer T cells (NKT) cells, and γδ T cells. We also review the current status of innate immune cell-based therapy for the treatment of osteosarcoma and potential future improvements based on the results of treatment of other types of tumors. Moreover, immune checkpoint inhibitors (ICPIs) represent a new frontier in cancer therapy and have shown a certain degree of therapeutic effects in osteosarcoma patients (15). Some immune checkpoints are not only expressed on T cells, but also on DCs, macrophages, NK cells, NKT cells, and γδ T cells; blocking these immune checkpoints reverses their anti-tumor activity in tumor immunity. Therefore, we detail the effects of immune checkpoint-inhibition on immune cells and the potential for synergy based on combining innate immune cell-based therapy with immune checkpoint manipulation for the treatment of osteosarcoma. In addition, as oncolytic virus (OV) therapy is known to induce an innate immune response, we also discuss the combinational potential of innate immune cell-based therapy and OVs.

## Dendritic Cells

DCs, which are professional antigen-presenting cells (APCs), take up and present antigens to naïve T cells, ultimately stimulating them to differentiate into tumor killers (16). Recently, a series of studies have shown that DCs can also activate innate immune cells with robust antitumor activity such as γδ T cells, cytokine-induced killer (CIK) cells (17–19).

**Figure 1:**
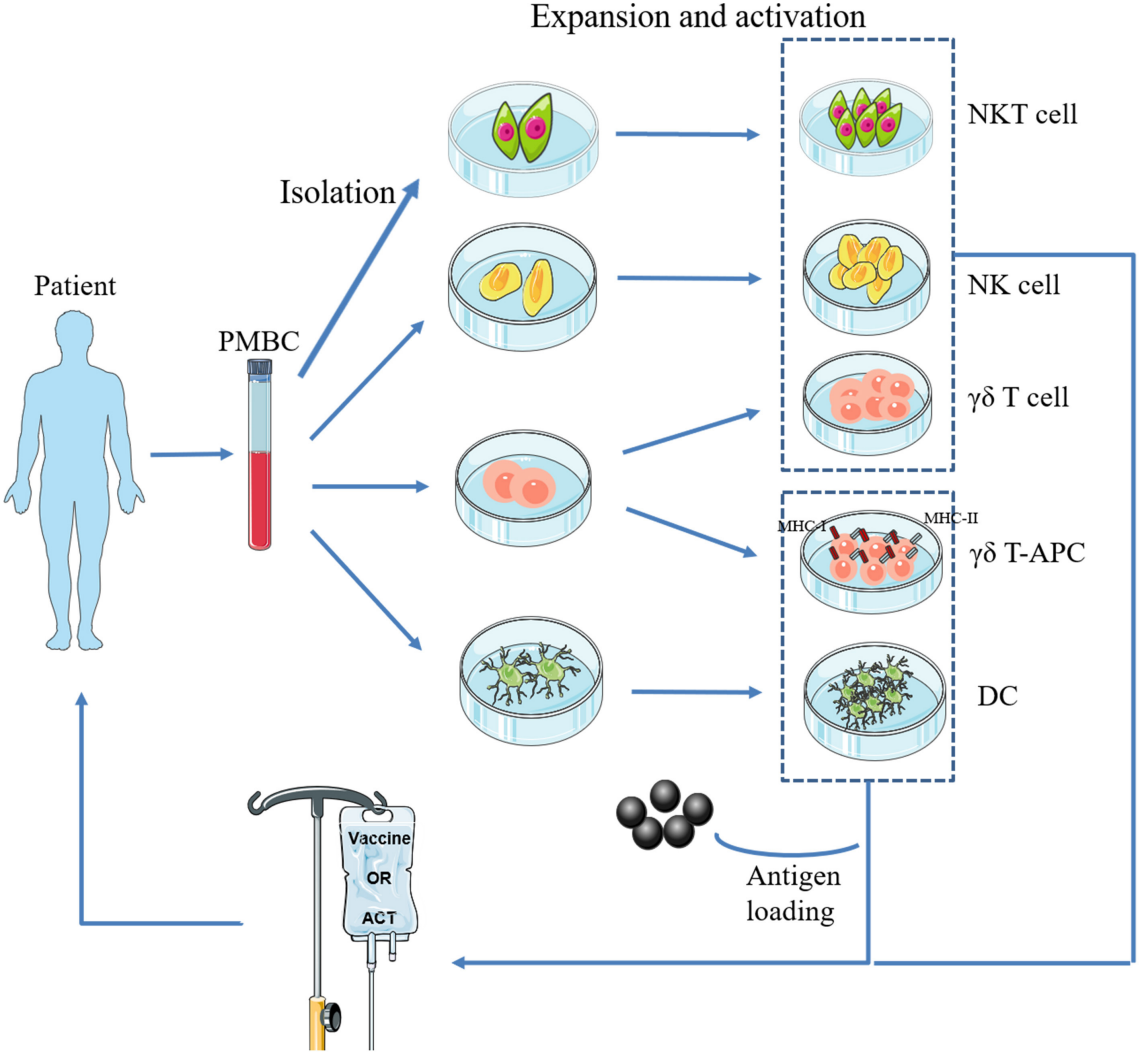
Basic procedure of adoptive transfer of innate immune cells. NKT cells, NK cells, γδ T cells, and DCs are isolated from a patient's PBMCs, expanded and activated *ex vivo*, and then infused back into the body. In particular, γδ-APC and DCs need to be loaded with tumor antigen(s).

However, established tumors always endeavor to reduce the availability of antigen presentation by APCs, resulting in immunosuppression, which disrupts the generation of antitumor immune responses (20, 21). In response, DC vaccines have been developed to bypass this mechanism. This procedure can be summarized as follows: DCs are isolated from peripheral blood mononuclear cells (PBMCs), matured, and loaded *ex vivo* with tumor antigens with defined cocktails, and then infused back into the patient (Figure 1). Theoretically, these antigen-activated DCs can successfully boost the immune response. Recent preclinical studies of osteosarcoma DC vaccines are listed in (Table 1). They can be classified into three major groups based on the protocols for loading various sources of antigens (33): (1) DCs co-cultured with peptides, proteins, or tumor-cell lysates; (2) DCs transfected with DNA, RNA coding for antigens, or total RNAs derived from tumor cells; and (3) fusions between DCs and devitalized tumor cells. Yu et al. (23, 24) tested the efficacy of osteosarcoma DC vaccines either fused with whole-tumor cell or transduced with total tumor RNA. Most immunized tumor-free rats acquired partial or complete protection from tumor challenge. In addition, vaccination induced tumor suppression in tumor-bearing mice (23, 24). Other studies tested the potential of combination therapy consisting of a DC vaccine and targeted drugs such as anti-transforming growth factor-β (TGF-β)/glucocorticoid-induced tumor necrosis factor receptor (GITR) antibodies (30, 32). The results of these studies showed that primary and metastatic tumor growth was inhibited. In addition, the tumor microenvironment (TME) was remodeled with reduced number of regulatory T lymphocytes (Tregs), reduced levels of immunosuppressive cytokines, and an increased number of CD8^+^ T lymphocytes (30, 32). However, DC vaccines were less effective for the treatment of osteosarcomas in clinical trials (34–36). For instance, only two out of 12 patients exhibited a strong anti-tumor immune response, and none exhibited any clinical effects, after receiving 3 weekly DC vaccine administrations (35). However, DC vaccines were well-tolerated in all the clinical trials.

**Table 1:** Pre-clinical studies of DC-based vaccines for osteosarcoma.

**Type of DC vaccine**	**Study type**	**Ancillary therapy**	**Effect**	**References**
Autologous DCs transfected with total tumor mRNA	*In vitro*	CIK cells	Effective osteosarcoma cytolysis	(19)
	*In vivo*	None	Induction of specific CTL responses, tumor rejection in 70% of vaccinated tumor-bearing rats, and development of long-term immunological memory to reject a subsequent tumor rechallenge	(22)
	*In vivo*	None	Induction of specific CTL responses, tumor rejection in 80% of vaccinated tumor-bearing rats and development of long-term immunological memory to reject a subsequent tumor rechallenge	(23)
Allogeneic DCs fused with tumor cells	*In vivo*	None	Protection from tumor challenge in 70% of pre-vaccinated rats and tumor rejection in 60% of tumor-bearing rats	(24)
	*In vitro*	None	Effective activation of T cells	(25)
Autologous DCs fused with tumor cells	*In vitro*	None	Effective activation of T cells	(26)
	*In vivo*	None	Atrophy or disappearance of tumor bodies and higher survival times and rates	(27)
Autologous DCs loaded with tumor cell lysate	*In vitro*	None	Increased induction of CTL activity	(28)
	*In vivo*	None	Increased number of CD8^+^ T lymphocytes in the metastatic areas, and reduced pulmonary metastases	(29)
	*In vivo*	Anti-TGF-β antibody		(30)
	*In vivo*	Anti-CTLA-4 antibody		(31)
	*In vivo*	Anti-GITR antibody	Increased number of CD8^+^ T lymphocytes in tumor tissue and serum, inhibition of primary tumor growth, and prolonged survival	(32)

Three explanations can be proposed for the lack of clinical benefits in patients. (1) Compromised quality and quantity of the immune effector cells in patients. Osteosarcoma patients commonly receive a full course of upfront chemotherapy, which may damage the innate and adaptive immune effectors and thus limit their availability and efficacy to respond to the increased antigen presentation. (2) Poor migration of effector cells to the tumor site, probably due to down-regulation of chemokine expression. (3) Other strong immunosuppressive mechanisms, for example, immune checkpoints on immune cells. An effective cancer vaccine should be able to overcome tumor-associated immune suppression and reinstate immune surveillance (37). Therefore, increasing the ratio of active effector cells to tumor target cells, enhancing the infiltration of the effectors, or remodeling the TME in combination with administering DC vaccines may enhance antigen presentation, immune response, and clinical efficacy.

## Macrophages

In normal bone biology, osteoclasts, which are highly specialized macrophages, are involved in bone resorption and have central functions in bone homeostasis (1). Macrophages in the vicinity of osteosarcoma cells are identified as tumor-associated macrophages (TAMs). They consist of a large variety of subpopulations, which were initially classified as anti-tumor M1-polarized macrophages and pro-tumor M2-polarized macrophages (38). TAMs infiltrate massively into osteosarcoma tissues and contribute to tumor progression through multiple pathways. In preclinical models, macrophages recruited by interleukin (IL)-34 released by osteosarcoma cells promoted tumor progression and the metastatic process (39). Han et al. (40) found that osteosarcoma patients with detectable metastasis at diagnosis have more TAMs in the primary site. Interestingly, TAMs occurred at a higher rate in osteosarcoma lung metastases than in the corresponding primary lesions and promoted lung metastasis and induced epithelial-mesenchymal transition in osteosarcoma by activating the cyclooxygenase (COX)-2/signal transducer and activator of transcription (STAT)-3 axis (40). Additionally, Han et al. revealed that the number of M2-TAMs was correlated with the frequency of suppressive T-cell immunoglobulin and mucin-domain containing-3 (TIM-3)^+^ programmed cell death 1 (PD-1)^+^ T lymphocytes in osteosarcoma patients (41). TIM-3/Gal9 interactions between T cells and monocytes have been shown to resulted in an immunosuppressive response (42). These results indicate that TAMs promote tumor growth by suppressing intra-tumor T-lymphocytes. However, several studies have reached different conclusions. A study by Buddingh et al. demonstrated that TAMs were associated with metastasis inhibition in high-grade osteosarcoma patients (43). This result was recently confirmed in orthotopic osteosarcoma mouse models (44). Moreover, a biopsy study revealed that a high level of CD163 (a marker of M2-polarized macrophages) was related to longer metastasis progression-free survival (MPFS), and CD68 (a marker for macrophages) exhibited a similar association (45). The possible reason may be that the polarization/phenotype and infiltration of TAMs change dynamically during tumor growth, and the current studies do not fully represent the whole dynamic process of TAMs in the TME.

Despite the contradictory roles of TAMs in the TME, three therapeutic strategies targeting TAMs have shown potential for treating osteosarcoma. (1) Preventing polarization of M1 macrophages to M2, or directly suppressing the M2 phenotype. Pharmacological therapy for the treatment of osteosarcoma using all-trans retinoic acid (46), resveratrol (47), and dihydroxy coumarins (48) has shown favorable results involving the suppression of M2-polarized macrophages. (2) Enhancing non-TAM macrophages recruitment. A study showed that upregulation of Secreted Protein, Acidic and Rich in Cysteine-like 1 (SPARCL1) protein induced osteosarcoma cells to secrete chemokine ligand 5, resulting in macrophage recruitment. The recruited macrophages exerted anti-tumor effects and inhibited osteosarcoma metastasis (49). (3) Activating macrophages. Mifamurtide, an immunoadjuvant currently approved for osteosarcoma therapy in the European Union, can activate the tumoricidal properties of macrophages and inhibit human osteosarcoma cell growth (50, 51). A report from the international Children's Oncology Group found that the addition of mifamurtide to chemotherapy significantly improved overall survival from 70 to 78% and resulted in a trend toward improved event-free survival (EFS) among patients with no signs of metastasis (52). Similar benefits were observed in patients with metastatic osteosarcomas, although the results were not statistically significant (53).

## Natural Killer Cells

NK cells express a repertoire of activating and inhibitory receptors (Table 2) that recognize altered expression of proteins on target cells, allowing for control of NK cell functions. After activation, they exhibit spontaneous cytolytic activity against cells undergoing malignant transformation (54). Recently, immunologists found that NK cells could stimulate DC recruitment into the TME, resulting in inhibition of tumor growth (55). Osteosarcoma patients had lower numbers of NK cells at the time of diagnosis compared to normal controls (56). After IL-2 administration and polychemotherapy, osteosarcoma patients had increased numbers, and increased activity, of NK cells in the blood, the magnitude of which strongly correlated with the clinical outcomes (57). These data indicate that NK cells have anti-tumor immune activity and play a role in immune surveillance in osteosarcoma patients. Importantly, osteosarcoma cell-surface molecules make osteosarcoma cells particularly susceptible to NK cell-mediated killing. CD54 and CD58 (both of which are adhesion molecules) are fully expressed on osteosarcoma cells, allowing for easy recognition by, and a strong association with, NK cells (58, 59). In addition, human leukocyte antigen (HLA) class I (a ligand for inhibitory receptors on NK cells) is typically downregulated (3), while major histocompatibility complex class I chain-related protein A/B (MICA/B) and UL16-binding protein (ULBP) (ligands for activating receptors on NK cells) (60, 61) are overexpressed on osteosarcoma cells, allowing for easy activation of NK cells.

**Table 2:** Activating and inhibitory receptors on human NK cells.

**Type**	**Receptors**	**Ligands**
Activating receptors	NKG2D	MICA/B, ULBP1–4
	CD94-NKG2C	HLA-E
	KIR2DL4	HLA-G
	KIR2DS1	HLA-C2
	KIR2DS2	HLA-C1
	KIR2DS3	Unknown
	KIR2DS4	HLA-A11
	KIR2DS5	Unknown
	KIR3DS1	HLA-Bw4
	NKp30	B7H6, BAT3, pp65 of HCMV, viral HAPfEMP1 of *Plasmodium falciparum*
	NKp46	Heparin, viral HA and HN
	NKp44	Viral HA and HN, PCNA, proteoglycans
	DNAM-1	CD112, CD155
Inhibitory receptors	KIR2DL1	HLA-C2
	KIR2DL2	HLA-C1
	KIR2DL3	HLA-C1
	KIR3DL1	HLA-Bw4
	KIR3DL2	HLA-A3, -A11
	NKR-P1A	LLTI
	CD94-NKG2A	HLA-E
	ILT2 (CD85j)	HLA-A, -B, -C, HLA-G1, HCMV UL18
	CD244(2B4)	CD244(2B4)

Treatment of patients with cells that have been isolated, manipulated, and expanded *ex vivo*, and then reinfused into the patient, is called adoptive cell therapy (ACT) (Figure 1). Infused immune cells migrate and infiltrate into the tumor site and mediate antitumor effects. There are three ancillary strategies to further improve the therapeutic effectiveness of adoptive NK cell transfer in osteosarcoma immunotherapy (Table 3). First, epigenetic drugs, such as histone deacetylase inhibitors (HDACi, e.g., valproic acid [VPA], entinostat) and DNA-methylation inhibitors (DNMTi, e.g., hydralazine) can increase the expression of ligands for activating receptors (MICA/B, ULBP, and CD155) or death receptors (Fas) on osteosarcoma cells, enhancing NK cell-mediated lysis (62, 63, 65). Another DNA-methylation inhibitor, decitabine, has been shown to enhance γδ T cell-mediated cytotoxicity by inducing ligands for activating receptors (natural killer group 2D, member D [NKG2D] ligands [NKG2DLs]) on osteosarcoma cells (12). Combining decitabine with the NK cells might be equally effective for treating osteosarcoma. Additionally, some traditional chemotherapeutic drugs (including doxorubicin, cisplatin, and gemcitabine) have been found to increase NK cell-activating ligand expression in tumors (71). Though similar studies in osteosarcoma are rare, chemotherapeutic drugs can modulate death receptors (DRs) on osteosarcoma cells, which may make them more sensitive to Fas-mediated NK cell cytotoxicity. For example, gemcitabine up-regulated cell-surface Fas expression and was effective in treating osteosarcoma lung metastases (72). Interestingly, treatment with cisplatin could not upregulate the cell-surface Fas antigen but it did sensitize human osteosarcoma cells to Fas-mediated apoptosis by down-regulating the expression of FLICE inhibitory protein long form (FLIP-L). Second, cytokine therapy can enhance the conjugate-forming capacity of NK cells to osteosarcoma targets by augmenting the expression of CD18 and CD2 (68) (both of which are adhesion molecules on NK cells), and intercellular adhesion molecule (ICAM)-1 (67) and fibronectin (69) (both of which are adhesion molecules on osteosarcoma cells). Interestingly, cytokine therapy can also increase the killing activity of NK cells. For instance, IL-15, the most promising NK cell-activating cytokine, can strongly enhance NK cell-mediated cytolytic activity toward chemotherapy-resistant osteosarcoma (60, 66). IL-2 can also strongly augment NK cell activity (73). It has been widely shown that, in neuroblastoma, IL-2 administration combined with immunotherapy (involving anti-GD2 antibody) enhanced NK cell proliferation and cytotoxicity (74), and showed promising results in clinical trials (75). Importantly, IL-2 aerosolization in dogs and mice with osteosarcoma lung metastasis similarly enhanced the local proliferation and cytotoxicity of NK cells and induced metastatic regression (76, 77). Third, monoclonal antibodies can target various receptors on NK cells to improve NK cell cytotoxicity. One approach is to develop a monoclonal antibody (mAb) to facilitate antibody-dependent cell-mediated cytotoxicity (ADCC) against osteosarcoma cells. Cetuximab, a mAb that targets epidermal growth factor receptor (EGFR) on target cells, with an Fc region that binds to CD16 on NK cells, increases NK-dependent lysis of EGFR-expressing osteosarcoma cell lines by enhancing ADCC (70). Another approach is to block the inhibitory NK cell receptors (such as NKG2A or KIR2DL-1,−2, and−3) using mAbs (78, 79). However, this approach has not been evaluated for treating osteosarcoma. Emerging evidence has shown promising strategies for osteosarcoma treatment, and carefully designed clinical trials may demonstrate the effectiveness of these therapies.

**Table 3:** Classification of immunomodulatory strategies for improving the killing effectiveness of adoptive NK cell transfer therapy against osteosarcoma.

**Immunomodulatory strategy**		**Mechanism**	**Study type**	**Comment**
Epigenetic drug	VPA	Augmented expression of MICA/B on tumor cells	*Ex vivo*	VPA sensitized human osteosarcoma cells to cytotoxicity of NK cells (62)
	Entinostat	Augmented expression of MICA/B, ULBP, and CD155 on tumor cells	*In vivo*	Entinostat failed to augment the efficacy of NK cell therapy in a nude mouse model of human osteosarcoma lung metastasis (63)
	Entinostat	Downregulation of the anti-apoptotic protein, c-FLIP, and increased levels of Fas within the membrane lipid rafts on tumor cells	*Ex vivo*	Entinostat sensitized osteosarcoma cells to NK cell-mediated apoptosis (64)
	VPA+ hydralazine	Augmented expression of MICA/B and Fas on tumor cells	*Ex vivo*	VPA combined with hydralazine enhanced the susceptibility of osteosarcoma cells to Fas- and NK cell-mediated cell death (65)
Cytokine	IL-15	Enhanced DNAM-1 and NKG2D signaling pathways	*Ex vivo*	IL-15 enhanced cytolytic activity against chemotherapy-resistant osteosarcoma cells (60)
	IL-15	Prevention of down-regulation of NKG2D on NK cells	*Ex vivo*	IL-15 reversed inhibition of NK cell-mediated cytolytic activity against osteosarcoma (66)
	IL-12+IFN-γ+IL-18	Enhanced expression of ICAM-I on HOS cells	*Ex vivo*	IL-12 enhanced NK-mediated cytolysis of HOS cells in the presence of IFN-γ and with IL-18 (67)
	IL-12+IL-2	Increased density of CD18 and CD2 molecules on NK cells	*Ex vivo*	A combination of IL-12 and IL-2 increased lytic activity against and binding to osteosarcoma cells (68)
	IL-17	Increased expression of fibronectin on U2 OS cells	*Ex vivo*	IL-17 enhanced NK cell-mediated adhesion and cell lysis activity against osteosarcoma (69)
Monoclonal antibody	Cetuximab	ADCC	*Ex vivo*	Cetuximab augmented cytolytic activity of resting NK cells, which was specifically directed toward osteosarcoma cells (70)

Genetic engineering of immune cells can endow them with additional antitumor specificity. For instance, transduction of precise and functionally active chimeric antigen receptors (CARs) into NK cells has led to stronger cytotoxicity toward osteosarcomas. A receptor designated NKG2D-DAP10-CD3ζ (comprising the NK cell- activating receptor NKG2D and two key signaling molecules, DAP10 and CD3ζ) was recently developed. Transduction with this chimeric receptor markedly increased NKG2D surface expression on NK cells and the transmission of activating signals. In a xenograft model of osteosarcoma, adoptive transfer of these CAR-NK cells significantly decreased the overall tumor burden (80). However, there are technical challenges to overcome to obtain sufficient numbers of functionally active NK cells from a patient's blood. The emergence of the human NK92 cell line consisting of activated NK cells may resolve the challenges faced by CAR-NK cell-based therapy, as NK92 cell line is relative ease in *ex vivo* large-scale expansion and effective receptor transfection (81). Adoptive transfer of NK-92 cells transduced to express various CARs was shown to cause tumor regression in various tumor xenografts (82, 83). CAR-NK-92 cell-based therapy is currently being evaluated in clinical trials for CD33^+^ acute myeloid leukemia (AML; NCT02944162) and CD7^+^ leukemia and lymphoma (NCT02742727). Therefore, utilizing NK-92 cell line for producing sufficient CAR-NK cells (e.g., NKG2D-DAP10-CD3ζ-transduced NK92 cells) to effectively target and eliminate osteosarcoma is a promising strategy that requires further evaluation. However, NK92 cell line must be irradiated before being infused into patients (81), which limits the survival and proliferation of NK cells—two key factors that are known to influence the efficacy of NK cell-based immunotherapy (84). In contrast, large-scale differentiation of human induced pluripotent stem cells (iPSCs) into NK cells (with phenotypic and functional similarities to NK cells isolated from peripheral blood) is relatively easy (85). After CAR transduction, the efficiency of NK cell production from iPSCs is similar to the efficiency of NK cell production from non-CAR-expressing iPSCs (86). Moreover, NK cells derived from human iPSCs that express CARs (CAR-iPSC-NK cells) have a typical NK cell phenotype. In a mouse xenograft model of ovarian cancer, CAR-PSC-NK cells (with a CAR comprising the NK cell-activating receptor NKG2D, the co-stimulatory domain 2B4 and the key signaling molecule CD3ζ) showed increased *in vivo* expansion and improved activity with less toxicity (87). CAR-iPSC-NK cells mediate their activity without requiring HLA matching; therefore, theoretically, they can also be used to treat other solid tumors including osteosarcoma. Recently, clustered regularly interspaced short palindromic repeats (CRISPR)/CRISPR-associated protein 9 (Cas9) technology has been used to edit CAR T cells (88). For example, knocking out immune checkpoints may protect CAR T cells from being exhausted (89). Knocking out αβ T-cell receptors (TCR) (88) or β2-microglobulin (β2M) (90) minimized the risks associated with “off-the-shelf” CAR T cells. Delivering a CAR gene to a specific locus, TCR α constant (TRAC), yielded therapeutic CAR T cells that were more potent (91). To achieve a robust anti-tumor effect, applying CRISPR/Cas9 technology to edit CAR-NK cells (e.g., by knocking out immune checkpoints) should be further investigated.

## Natural Killer T Cells

NKT cells express molecular markers of both NK cells (e.g., NK1.1, Ly49, NKRs, and KIRs) and T cells (e.g., αβ TCR, CD44, CD69, and CD122). In tumor immunity, activated NKT cells are able to kill tumors via different NK and T cell-associated mechanisms (92, 93). In addition, high numbers of tumor-infiltrating NKT cells correlated with good clinical outcomes in cancer patients (94, 95). However, in some tumor types, the number of NKT cells was higher compared to the number in normal tissue (94, 96). Further studies focusing on function and phenotype of tumor-infiltrating NKT cells showed that they expressed fewer activating receptors and produced lower amounts of pro-inflammatory cytokines compared with para-carcinoma tissues (97, 98).

A similar contradictory function of NKT cells in osteosarcoma immunity was observed. One research group found that NKT cells purified from human PBMCs and expanded *ex vivo* enhanced osteosarcoma cell death induced by standard chemotherapy (doxorubicin, cisplatin, and methotrexate) (99). In contrast, other researchers found that tumor-infiltrating NKT cells had a negative regulatory role, involving suppression of CTL function (100). A hypothetical model of NKT cell functional transformation in osteosarcoma is as follows: during the early tumor stage, the NKT cell subpopulation exerts effective antitumor immune responses against tumors. However, during tumor progression, NKT cells become overstimulated and anergic, and they finally transform, contributing to tumor immune escape (101).

Two major aspects of current NKT cell therapeutic strategies should be carefully considered in light of this hypothetical model. (1) *in situ* expansion and activation of NKT cells in early tumor stages or adoptive transfer of *ex vivo* expanded and activated autologous NKT cells into patients (Figure 1). α-galactosylceramide (GalCer) or α-GalCer-pulsed autologous DCs is a common strategy to activate NKT cells *in vivo* or *ex vivo* (102, 103). Recent studies found that iPSCs might be more effective at amplifying the numbers of autologous NKT cells (104, 105). (2) Skewing of pro-tumor NKT cells toward anti-tumor subtypes in advanced tumor stages. The addition of IL-12 (106) or chemical modification of α-GalCer (107) skewed the conventional α-GalCer-produced TH1- and TH2-associated cytokines toward only TH1-associated cytokine production. These data indicate that pro-tumor NKT cells were transformed to anti-tumor subtypes following this intervention.

## γδ T Cells

It has been found that γδ T cells can mediate effective antitumor immune responses. In a methylcholanthrene (MCA)-induced sarcoma model, γδ T cell-deficient mice had an increased incidence of tumor development (108). Preclinical studies found that γδ T cells could directly kill malignant cells through the generation of cytokines (tumor necrosis factor [TNF]-α and interferon [IFN]-γ), upregulation of activating receptors or their ligands (Fas-L, NKG2D, TRAIL, and TNF), expression of CD16 for ADCC, and release of granzymes and perforin (109). Recent studies indicated that, in the short-term, γδ T cells possess phenotypic characteristics of DCs after activation by phosphoantigens (110). The effect of priming a strong CD8^+^ T cell-mediated anti-tumor response using peptide-pulsed γδ T cells was even more powerful than the effect induced by DCs (111, 112).

The main advantages of adoptive γδ T cell transfer immunotherapy (Figure 1) are as follows: (1) γδ T cells can infiltrate the TME (113, 114); (2) they exert cytotoxic activity against cancer cells in an HLA-independent manner; and (3) they can be expanded and activated *ex vivo* by simple yet effective protocols (115). Kato et al. (116) initially reported the ability of γδ T cells to directly recognize and kill osteosarcoma cell lines NY, SAOS2, and OST. However, these cell lines were only moderately susceptible to γδ T cell cytotoxicity. Therefore, later studies have focused on adjuvant therapies to potentiate the immunosensitivity of osteosarcoma cells to γδ T cells (Table 4). Zoledronate (ZOL) significantly reduces skeletal complications in patients with bone metastases from solid tumors (120) and inhibits osteosarcoma growth (121). Our group and other researchers demonstrated that ZOL could also enhance the anti-osteosarcoma activity of γδ T cells (14, 117). However, the specific mechanisms have not been elucidated and a high dose of ZOL is necessary to achieve this effect, while the ZOL concentration in the blood declines rapidly (122). Recently, a study by our group found that a ZOL-related mechanism was associated with increased accumulation of mevalonate pathway intermediates (11). We also found that VPA (the HDACi) and ZOL had a synergistic effect on the enhancement of γδ T cell-mediated cytotoxicity against osteosarcoma cells by facilitating the accumulation of mevalonate pathway intermediates (11). More usefully, this combination therapy reduced the ZOL dose required in adoptive γδ T cell transfer immunotherapy, facilitating its clinical application (11). In addition, the expression of human epidermal growth factor receptor 2 (Her-2) was associated with tumor progression and poor prognosis in osteosarcoma patients (123). However, no therapeutic effectiveness was observed pre-clinically or clinically for trastuzumab (an anti-Her-2 monoclonal antibody)-driven osteosarcoma therapy (124). However, Liu et al. reported that trastuzumab aided γδ T cell-mediated lysis of osteosarcoma cells by enhancing ADCC (13), suggesting a promising novel combination regimen to treat osteosarcoma. Additionally, it was reported that bispecific antibodies could enhance the cytotoxicity of γδ T cells. For example, a research group designed a bispecific antibody, Her2/Vγ9, that binds to Vγ9 on γδ T cells and Her-2 on pancreatic tumor cells (125). Infusion of this novel bispecific antibody improved recognition and binding between adoptively transferred γδ T cells and tumor cells, significantly reducing pancreatic tumor growth in mouse models. This result suggests that Her2/Vγ9 antibody might promote the capacity of γδ T cells to lyse osteosarcoma cells to a greater extent than Her2 antibody. Furthermore, IFN-γ and decitabine (a DNA demethylation drug) increased γδ T cell cytotoxicity against osteosarcoma cells by increasing the expression of Fas and NKG2DLs on tumor cell surfaces (12, 118).

**Table 4:** Chronological summary of studies on γδ T cell therapy against osteosarcoma.

**References**	**Ancillary therapy**	**Study type**	**Cell type and source**	**Mechanism**	**Result**
Muraro et al. (117)	ZOL + IL-2	*In vitro*	γδ T cells from HD	Unknown	Potent anti-tumor activity of γδ T cells against osteosarcoma cell lines
Li et al. (118)	IFN-γ	*In vitro*	γδ T cells from HD	Up-regulated expression of Fas on osteosarcoma cell lines	Enhanced cytotoxic effect of γδ T cells against osteosarcoma cell lines
Li et al. (14)	ZOL	*In vitro*	Vγ9Vδ2 T cells from OP and HD	TCR-mediated and partly NKG2D-mediated granule exocytose and TRAIL pathways	Potent anti-tumor activity of Vγ9Vδ2 T cells
Liu et al. (13)	Trastuzumab + ZOL	*In vitro*	Vγ9Vδ2 T cells from HD	ADCC	More efficient ability of Vγ9Vδ2 T cells to recognize and lyse osteosarcoma cell lines.
Li et al. (119)	Celastrol	*In vitro*	γδ T cells from OP and HD	Up-regulation of death receptors 4/5 on osteosarcoma cell lines	Increased osteosarcoma cell lysis by γδ T cells
Wang et al. (11)	ZOL+VPA	*In vivo*	γδ T cells from OP and HD	Increased accumulation of the mevalonate pathway intermediates in osteosarcoma primary cells and cell lines	Enhanced γδ T cell migration and antitumor effect.
Wang et al. (12)	Decitabine	*In vivo*	γδ T cells from OP	Increased expression of NKG2DLs on osteosarcoma cell lines	Enhanced antitumor effect of combination therapy of γδ T cell infusion and decitabine administration

*HD, healthy donors; OP, osteosarcoma patients*.

Recent achievements in cell engineering and further studies of γδ T cell physiology have provided an improved foundation for improving γδ T cell-based immunotherapies. Three potential perspectives related to potentiating the cytotoxicity of γδ T cells are as follows. (1) T cells transduced with TCRs that specifically target the NY-ESO-1 antigen on tumors are called NY-ESO-1-specific TCR-engineered T cells. These cells can be activated upon encountering NY-ESO-1 antigens presented by HLA molecules and they then specifically target and kill tumor cells. Adoptive transfer of NY-ESO-1-specific TCR-engineered T cells represents a potentially effective therapeutic approach for the treatment of osteosarcoma (126). However, introduction of α/β chains has the potential to result in mispairing with endogenous α/β TCR chains, resulting in mixed TCR dimers with unknown specificities, which can lead to adverse complications such as autoimmune responses and toxicity. However, previous studies showed that α and β TCR chains could not form heterodimers with γ and δ TCR chains when transduced into γδ T cells (127). Meanwhile, αβ TCR-transduced γδ T cells exhibited high levels of cytokine release and cytotoxic activity (127, 128). Therefore, using NY-ESO-1-specific αβ TCR-transduced γδ T cells to treat osteosarcoma may be a safe and effective strategy. (2) γδ T cells may be ideal candidates for cell vaccine manufacturing (Figure 1). The advantages of γδ T cell vaccines compared to DC vaccines are as follows (129): first, obtaining and expanding γδ T cells to create an unlimited number is easy, economical, and highly selective; second, γδ T cell vaccines display excellent survival during *ex vivo* preparation, allowing for possible freezing for storage and shipment to cancer clinics in large quantities; third, the status of γδ T cells is uniform (effector-memory), while DCs remain heterogeneous (immature-mature-exhausted); finally, γδ T cells have functional uniformity with stable induction of primarily pro-inflammatory responses. (3) Mechanistic target of rapamycin (mTOR) is important for regulating T cell metabolism and function. Recent studies have demonstrated the important role of mTOR in γδ T cells. Rapamycin (the US Food and Drug Administration [FDA]-approved mTOR inhibitor) increased the yield and durability of the elicited γδ T cell response (130). Later studies demonstrated that the immune stimulatory effects of rapamycin are mediated by boosting perforin release, enhancing tumor core infiltration, and upregulating NKG2D and TNF-α (131, 132). Therefore, it is conceivable that inhibition of mTOR receptors could contribute to γδ T cell-mediated osteosarcoma cell killing.

## Combination Therapy With Immune Checkpoint Inhibitors

Immune checkpoint molecules are key modulators of the anti-tumor T cell immune response by a narrow definition. Actually, multiple immune checkpoint molecules are also expressed on innate immune cells, which function as immunomodulators. Their interactions activate either inhibitory or activating immune signaling pathways. Indeed, metabolic pathways play a critical role in the functional modulation of immune cells and could, by extension, be considered as immune checkpoints. Here, we focus on the inhibitory immune checkpoints that influence adaptive and innate immune cells. Blocking inhibitory checkpoints can reverse the exhaustion state of immune cells and inhibit tumor growth. Importantly, one clinical trial demonstrated the immune response to ICPIs in osteosarcoma patients (15) and rational combinations of immunotherapies, particularly those involving ICPIs, have demonstrated increased efficacy in cancer patients (133). Therefore, ICPIs have the potential to improve efficacy of innate immune cell-based therapy for osteosarcoma.

### Programmed Cell Death 1

Programmed cell death 1 (PD-1) is a receptor expressed on the surface of T lymphocytes, and innate immune cells. PD-1 binds a specific ligand, programmed cell death ligand 1 (PD-L1), which is expressed on several types of malignant cells and APCs in tumor foci. It is widely accepted that PD-1 is an exhaustion marker for CTL (134), which is the main anti-tumor effector cell during checkpoint blockade therapy. A study aiming to find predictors of DC vaccine responses showed that glioblastoma patients with tumor-infiltrating lymphocytes (TILs) with a higher PD-1^+^/CD8^+^ ratio had worse prognosis (135). These data indicated that DC vaccine-primed CD8^+^ T cells became exhausted via the PD-1-PD-L1 axis, which is one of the reasons that DC vaccines have showed unsatisfactory results in osteosarcoma patients. This obstacle might be overcome by ICPIs. On the other hand, evidence indicates that a mechanism of acquired resistance to ICPIs involved alterations in antigen presentation (136). This problem can be solved by growing DC vaccines *ex vivo*. Therefore, PD-1 inhibitors and DC vaccines have complementary roles regarding antitumor efficacy (37, 137, 138). For instance, an *ex vivo* study demonstrated that anti- PD-1 treatment enhanced T-cell responses induced by DC vaccines fused with myeloma cells (137). Furthermore, in melanoma-bearing mice, anti-PD-1 treatment increased the function and infiltration of TILs induced by DC vaccines, and augmented anti-tumor activity (138). Currently, there are ongoing phase I/II clinical trials studying the effects of different types of DC vaccines combined with nivolumab (a mAb that blocks PD-1) for the treatment of glioma (NCT02529072), glioblastoma multiforme (NCT03014804, NCT02529072), and solid tumors (NCT02775292).

Interestingly, some cancer types exhibit low MHC I expression and/or neoantigen burden, which renders them resistant to recognition by CD8^+^ T cells, but sensitive to PD-1/PD-L1 axis blockade (139). This suggests that other immune cell types might also be suppressed by this axis. PD-1 expression on NK cells has been detected in cancer patients, including those with Kaposi sarcoma and ovarian carcinoma (140, 141). Preclinical observations showed that PD-L1 upregulation on several types of tumor cells or DCs suppressed NK cell-mediated tumor cell lysis, and blockade of PD-1 restored NK cell anti-tumor activity and inhibited tumor growth (141, 142). Importantly, a recent clinical study demonstrated that blocking PD-1 and PD-L1 elicited a strong NK cell response that was indispensable for the full therapeutic effects of immunotherapy (139). These data suggested the importance of the PD-1/PD-L1 axis in inhibiting NK cell responses *in vivo* and revealed that NK cells mediate the effect of PD-1/PD-L1 blockade immunotherapy. In addition, combination therapy consisting of NK cell transfusion and PD-1 blockade resulted in more potent cytolytic activity against tumor cells *in vitro* (142, 143). Unfortunately, a phase II clinical trial evaluating the effects of pembrolizumab, an anti-PD1 mAb, on the NK cell exhaustion phenotype in patients with unresectable stage III/IV melanoma (NCT03241927) has just been terminated because of difficult participant enrollment. Otherwise, this trial can aid in understanding how NK cell activity and exhaustion interplay with PD-1 expression and function, and it can lead to the development of more effective combination therapies.

PD-1^+^ TAMs, which exhibited an M2-like surface profile and M2-like functional characteristics and suppressed CD8^+^ (144) and CD4^+^ (145) T cell function, were detected in human cancers. In a human LM7 osteosarcoma mouse model, macrophages in lung metastases highly expressed PD-1 (146). PD-1 blockade significantly decreased the number of osteosarcoma lung nodules by increasing the macrophage tumor infiltration and polarization from M2 to M1 (146). Other research showed that PD-1 levels on tumor-infiltrating DCs were increased during tumor progression, and these DCs responded poorly to tumor antigens, and suppressed T cell activity and infiltration (147). In a murine model of ovarian cancer, targeting PD-1 on DCs significantly enhanced antigen-specific T cell responses and slowed tumor growth (147).

PD-1/PD-L1 expression was increased in osteosarcoma patients and correlated with poor prognosis (148, 149). In preclinical trials, PD-1 blockade resulted in anti-metastatic effects in osteosarcoma murine models (150, 151). However, PD-1 blockade was ineffective in an orthotopic osteosarcoma model (152). In addition, data from a multicenter, two-cohort, single-arm, open-label, phase II trial revealed that the effect of pembrolizumab (a PD-1 inhibitor) on osteosarcoma patients was poor (only one [5%] of 22 patients showed a partial response) (15). Therefore, it was urgent to improve the therapeutic effects of PD1/PDL-1 inhibitors. Recently, oncologists defined tumors lacking various inflammatory immune cell infiltration as “cold tumors,” and the opposite as “hot tumors” (153). Hot tumors are more susceptive to ICPIs. However, osteosarcomas are relatively “cold tumors.” A potential approach for reducing acquired resistance to ICPIs is turning a cold tumor into a hot tumor, resulting in enhanced infiltration of inflammatory immune cells (both adaptive and innate immune cells) into the tumor (154, 155). Therefore, further investigation of combination therapy involving an ICPI with an innate immune cell-based therapy (such as ACT and vaccines) for the treatment of osteosarcoma may be of value.

### Cytotoxic T-Lymphocyte-Associated Protein 4

Cytotoxic T-lymphocyte-associated protein 4 (CTLA-4) is another major immune checkpoint molecule on T cells induced by activation. CTLA-4 negatively regulates T cell function (156), and blocking CTLA-4 can reactivate T cells and enhance the efficacy of osteosarcoma vaccines. For example, in a C3H murine osteosarcoma model, tumor lysate-pulsed DCs with CTLA-4 blockade prevented lung tumor metastasis (31). Furthermore, a clinical study on the combined effects of a synthetic mRNA-electroporated DC vaccine and ipilimumab (an anti-CTLA-4 mAb) for patients with pretreated advanced melanoma showed a 6-month disease control rate of 51% and a promising overall response rate of 38% (eight complete and seven partial responses) (157). These results greatly increased interest in combination therapies involving vaccines and ICPIs. However, studies focusing on CTLA-4 expression on NK cells are scarce. CTLA-4 was detected on tumor-infiltrating NK cells in tumor-bearing mice and was closely associated with the inhibition of DC-induced IFN-γ production by NK cells (158). No studies have evaluated the expression of CTLA-4 on human NK cells. However, CTLA-4 may exist on human NK cells and may modulate their effector functions in cancer immunity.

CTLA-4 is significantly associated with carcinogenesis of osteosarcomas, which provides a potential therapeutic target (159). In a preclinical study, co-inhibition of CTLA-4 and PD-L1 resulted in complete control of metastatic osteosarcoma (151). Combined therapy involving anti-CTLA-4 antibody and a DC vaccine led to a similar outcome (31). Future studies should explore the possibility of combining anti-CTLA-4 mAb and NK cell-based therapy.

### T-cell Immunoglobulin and Mucin-Domain Containing-3

T-cell immunoglobulin and mucin-domain containing-3 (TIM-3) is expressed by innate and adaptive immune cells. Importantly, all TIM-3^+^ T cells in cancer patients co-express PD-1 (160). The current view is that CTLs with TIM-3-PD-1 co-expression are functionally more “exhausted” than those that express PD-1 alone (161, 162). Therefore, a DC vaccine combined with co-inhibition of TIM-3 and PD-1 may further prime T cells and maintain their cytotoxicity against malignant cells.

The inhibitory function of TIM-3 on innate immune cells (including NK cells, NKT cells, DCs, and macrophages) is consistent with its function on T cells (163). TIM-3 expression on peripheral NK cells correlated with their exhausted phenotype and predicted poor prognosis of patients with advanced melanoma and lung adenocarcinoma (164–166). Blockade of TIM-3 on NK cells from these patients increased NK cell-mediated cytotoxicity and IFN-γ production. Interestingly, researchers found that co-expression of TIM-3 and PD-1 is a marker of functionally exhausted NK cells in advanced tumors, as is the case for T cells (167). TIM-3 expression on macrophages is associated with inhibitory function in inflammatory diseases and cancers (168–170). For instance, in hepatocellular carcinoma, TIM-3 expression on TAMs was significantly enhanced by tumor-derived signals, which caused the macrophages to undergo alternative activation and inhibited CTL activation. Subsequent interference with TIM-3 on the TAMs successfully suppressed hepatocellular carcinoma growth (170). Recent studies showed that M1 macrophages had low expression of TIM-3, providing further evidence of its negative regulatory function in macrophages. In DCs, TIM-3 inhibits DC activation and maturation via the Btk-c-Src signaling pathway (171). In the TME, the interaction between TIM-3 and high-mobility group box 1 (HMGB1) prevented activation of tumor associated DCs by impeding sense of immunogenic nucleic acids, thereby suppressing anti-tumor responses (172). In γδ T cells, TIM-3 served as an exhaustion marker and protected the human body from inflammatory attack in different diseases (173, 174). Its role in tumor infiltrating γδ T cells has not been characterized.

Co-blocking CTLA-4 and PD-1 led to synergistic anti-tumor effects (175, 176). Interestingly, anti-CTLA-4 antibody showed a unique curative effect in anti-PD-1-resistant cancer (177). These results indicate that TIM-3 plays an essential role in tumor immunity. Therefore, TIM-3 is a candidate target for improving the effect of innate immune cell-based therapy.

### CD39/CD73 and Adenosine Receptors

**Figure 2:**
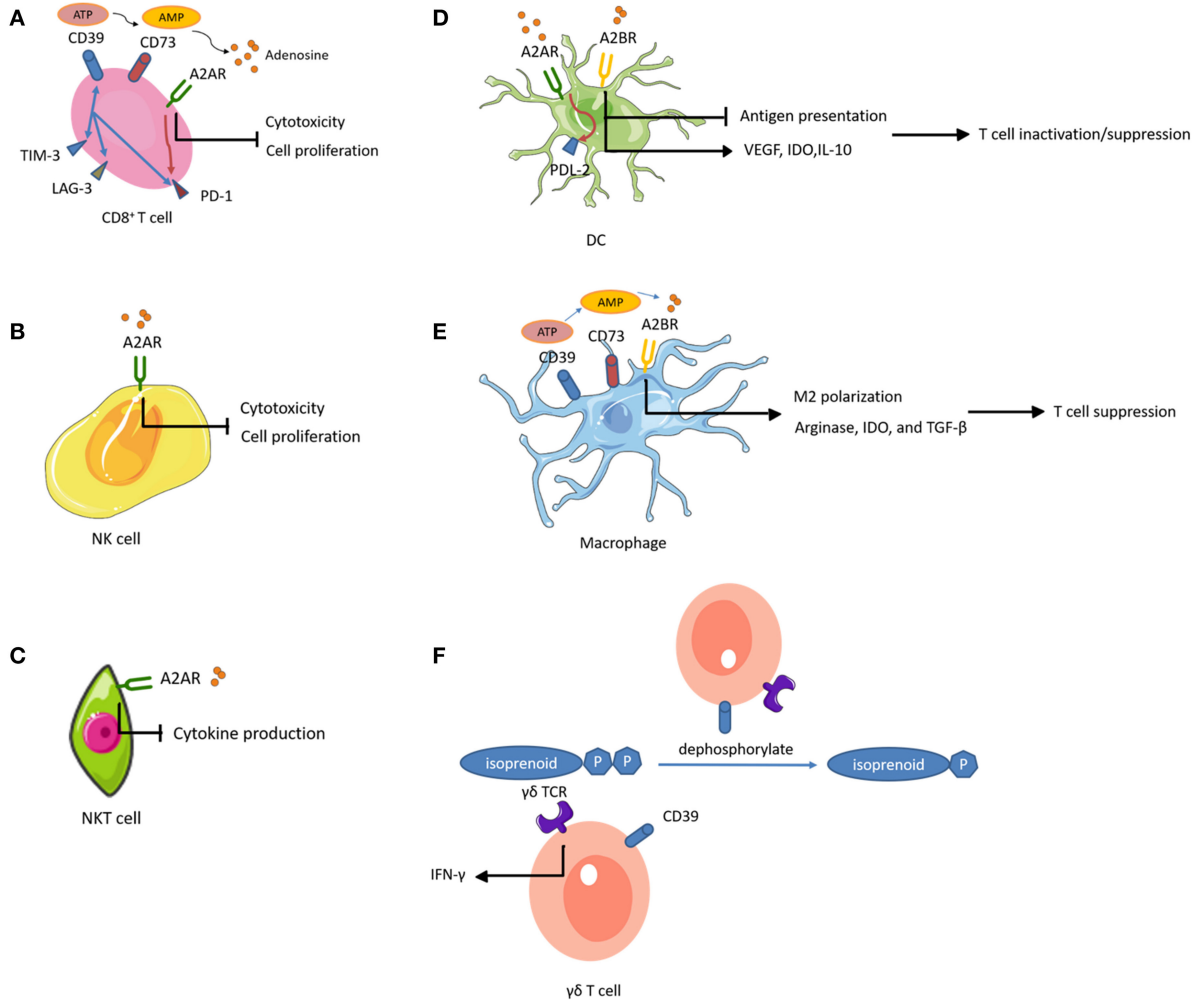
Adenosine-mediated immunosuppression of immune cells. Expression of CD39 and CD73 generates adenosine, an immunosuppressive metabolite. Activation of adenosine receptors (ARs) suppresses the proliferation and effector functions of cytotoxic lymphocytes, and promotes polarization toward exhausted or immunosuppressive function. **(A)** CD39^+^ CD8^+^ T cells highly express other inhibitory immune checkpoints such as PD-1, TIM-3, and lymphocyte activating 3 (LAG-3). **(A,B)** On CD8^+^ T cells and NK cells, A2AR activation inhibits their proliferation. **(A–C)** On CD8^+^ T cells, NK cells, and NKT cells, A2AR activation impairs their cytotoxic potential. **(A,D)** A2AR signal path on CD8^+^ T cells and DCs promotes the expression of other inhibitory immune checkpoints. **(A,E)** CD39 and CD73 expression on CD8^+^ T cells and macrophages contributes to adenosine accumulation. **(D)** On DCs, A2BR stimulation impairs DC antigen presentation and subsequent T cell priming while inducing VEGF, IDO, and IL-10 secretion and subsequent T cell suppression. **(E)** Activation of A2BR on macrophages favors M2 phenotype polarization and induces arginase, IDO, and TGF-β, mediating T cell suppression. **(F)** The ecto-ATPase CD39 inactivates isoprenoid-derived Vγ9Vδ2 T cell phosphoantigens.

In the TME, ATP conversion to ADP and/or AMP occurs in the presence of CD39 (also known as NTPDase 1), while CD73 (also known as 5′-NT) dephosphorylates AMP to adenosine. Accumulated extracellular adenosine exerts regulatory functions by binding to one of four adenosine receptors (ARs), A1R, A2AR, A2BR, and A3R (Figure 2).

A2AR activation increased cell-surface expression of PD-1 and CTLA-4 on T cells and inhibited proliferation and pro-inflammatory cytokine production (178). Similarly, a recent study showed that tumor-infiltrating CD8^+^ T cells expressed high levels of CD39 and exhibited an exhausted phenotype with impaired production of cytokines and high expression of inhibitory receptors (179). These observations suggested that CD39 was an immune checkpoint that could be targeted to restore the T cell immune response against tumors. In addition, genetic ablation or therapeutic inhibition of CD73 or AR improved the effector functions and infiltration of CTLs, and significantly reduced tumor growth (180–182). Importantly, these interventions augmented the efficacy of adoptive T cell anticancer therapy against ACT-resistant tumors (183, 184). These results indicated the potential to improve the efficacy of vaccines by inhibiting the adenosinergic pathway. Intravenous administration of CD73-specific small interfering RNA (siRNA)-loaded chitosan-lactate nanoparticles (ChLa NPs) potentiated the antitumor effects of a DC vaccine in 4T1 breast cancer-bearing mice, with augmented CTL effector function, improved T cell proliferations, and increased production of inflammatory cytokines (185). Similarly, another study demonstrated that co-targeting of A2AR and CD73 in conjunction with a DC vaccine successfully reduced tumor growth, prolonged survival, and enhanced specific antitumor immune responses in the same mouse model of breast cancer (186).

Notably, A2AR is abundantly expressed on NK cells (at a 5-fold higher level, at the mRNA level, compared to that in T cells), and A2AR activation inhibited NK cell cytotoxicity and proliferation in several tumors (187–189). A recent study found that co-inhibition of A2AR and PD-1 in a B16F10 lung metastasis model resulted in a therapeutic effect that was more dependent on infiltrating NK cells than T cells (190). These findings indicate an important role of A2AR regarding NK cell function in tumor immunity. In addition, antagonism of A2AR reduced the percentage of CD56^bright^ NK cells in favor of accumulation of mature CD56^dim^ NK cells with high cytotoxic activity (191). This suggested that A2AR antagonism could enhance adoptive NK cell immunotherapy. Adenosine-differentiated DCs displayed high levels of tolerogenic molecules (VEGF and indoleamine 2,3-dioxygenase [IDO]) and anti- inflammatory cytokines (IL-10), which impaired the DC antigen presenting function and subsequent T cell priming, resulting in accelerated tumor growth in mice (192, 193). Selective inhibition of A2BR improved DC activation and chemokine release, and subsequently increased T cell infiltration and adaptive responses in mice, resulting in reduced growth of carcinomas (194). Moreover, activation of the A2AR pathway in DCs increased the expression of programmed cell death 1 ligand 2 (PDL2, a ligand for the inhibitory receptor PD1), which directly inactivated effector T cells (195). Similarly, A2BR plays a prominent role in M2 polarization of macrophages (196). Macrophages differentiated in the presence of adenosine expressed arginase, IDO, and TGF-β, and had limited T cell stimulatory activity (196). Additionally, TAMs expressing CD39 and CD73 contributed to tumor growth through the production of adenosine (197, 198). Studies of the effects of adenosine-related molecules on γδ T cells are sparse. Upregulation of CD39 on human Vγ9Vδ2 T cells directly abrogated the γδ TCR agonistic activity of phosphoantigens (199). Through this pathway, CD39 reduced Vγ9Vδ2 T cell activation and IFN-γ production. This study revealed a previously unrecognized immunoregulatory function of CD39, which is independent of the adenosinergic pathway. A2AR activation also increased anti-inflammatory cytokine production in NKT cells, indicating that A2AR played a negative immune regulatory role in NKT cells (200).

Recent studies showed that intratumoral hypoxia and hypoxia inducible factor-1α (HIF-1α)-dependent pathways up-regulated the tandem activities of CD39 and CD73, leading to adenosine accumulation in the TME and tumor immune escape (201, 202). Adenosinergic pathways have not been characterized in osteosarcoma. However, studies have shown that hypoxia contributed to human osteosarcoma progression (203). It is conceivable that hypoxia-mediated tumor protection is dependent on adenosinergic pathway-mediated immunosuppression. Therefore, targeting CD39, CD73, and ARs has the potential to reinstate osteosarcoma immunity and improve immunosensitivity to innate immune cell-based immunotherapy.

### Clinical Studies of Innate Immune Cell-Based Immunotherapy and Immune Checkpoint Inhibitors

In this section, we mainly discuss the results of major clinical studies and ongoing clinical trials for treatment of osteosarcoma on innate immune cell-based immunotherapy and ICPIs for the treatment of osteosarcoma. As discussed above, the results of the initial clinical trials of DC vaccines were unsatisfactory (34–36), possibly due to tumor-associated immune suppression. A recent clinical trial (NCT01803152) has reported some improvements. The DC vaccine used was similar to the previous study (34–36), but the vaccine was combined with gemcitabine, which inhibits myeloid-derived suppressor cells (MDSCs) that play a vital role in tumor-associated immune suppression. In the field of innate cell infusion, NK cells are at the forefront. In an early clinical study, NK92 cells were infused into a patient with advanced osteosarcoma, though no treatment response was observed (204). More trial participants are required. We found several ongoing studies of expanded, activated haploidentical NK cell infusions for the treatment of sarcomas (these studies are summarized in Table 5), which should provide information on the effectiveness and safety of this approach. Only one clinical study published results regarding the curative effects of ICPIs for the treatment of osteosarcoma, which showed a 5% response rate to pembrolizumab (a PD-1 inhibitor) (15). Multiple clinical trials targeting PD-1 and/or CTLA-4 are ongoing (Table 5), and we expect an improved curative effect, which will provide a foundation for combination regimens involving targeting PD-1 and/or CTLA-4 along with innate immune cell-based immunotherapy.

**Table 5:** Clinical trials of DC vaccination, cell infusion, and ICPIs for treating osteosarcoma.

**Intervention**	**Ancillary therapy**	**Trial phase**	**Status**	**References**
Autologous DCs loaded with tumor cell lysates	None	I	Unknown	(35)^*^
	None	I/II	Unknown	(36)^*^
	Gemcitabine	I	Recruiting	NCT01803152
Autologous DCs loaded with TAAs or TAA-derived peptides (MAGE-A1, MAGE-A3, NY-ESO-1)	Decitabine	I/II	Completed	NCT01241162
Pembrolizumab (targeting PD-1)	None	II	Active, not recruiting	NCT02301039
SHR1020 (targeting PD-1)	Apatinib	II	Active, not recruiting	NCT03359018
Nivolumab (targeting PD-1) with ipilimumab (targeting CTLA-4)	None	II	Not yet recruiting	NCT02982486
Nivolumab with or without ipilimumab	None	I/II	Recruiting	NCT02304458
	None	II	Suspended	NCT02500797
NK cell infusion	None	I/II	Unknown	NCT02409576
	Haploidentical stem cell transplantation	II	Active, not recruiting	NCT01807468
	Hematopoietic cell transplantation	II	Recruiting	NCT02100891

*TAA, tumor-associated antigen*.

* *These studies were not found in ClinicalTrials.gov*.

## Combination Therapy With Oncolytic Viruses

Oncolytic viruses (OVs) are emerging as a novel therapeutic class, which selectively replicate in and lyse cancer cells without harming normal cells. Like chemotherapy and radiotherapy, the therapeutic outcomes of OVs are determined not only by direct cancer cell lysis, but also by immune activation (205). Here, we mainly discuss the innate immune responses induced by OVs.

Virus-infected cancer cells tend to down-regulate their MHC-I molecules making themselves more sensitive to NK cells (206). In this regard, several studies have been conducted to examine the anti-tumor effect of the combination of NK cells with OVs. As expected, combination therapy showed an additive or synergistic anti-tumor effect (207, 208). In addition, OV infection can lead to increased tumor infiltration of M1 type macrophages and NK cells (209, 210). Furthermore, infected cells can trigger a Toll-like receptor response due to the expression of pathogen-associated molecular patterns (PAMPs) on the cell surface or due to detection by intracellular components of Toll-like receptors (211). Additionally, OV infection can cause the exposure of calreticulin, HMGB-1, nucleic acids, and type I IFNs (212), and the induction of immunogenic cell death (213), which are essential ligands and innate immune sensing pathways for activation of DCs and macrophages (7). Oncolysis by OVs could also cause the release of tumor associated/specific antigens that are then cross-presented by DCs, ultimately eliciting an adaptive immune response against the tumor (214, 215). Some OVs, such as reovirus (216) and M protein mutant vesicular stomatitis virus (DeltaM51-VSV) (217), can directly activate DCs and facilitate their antigen presentation function.

## Conclusion

In view of the recent insights into the biology and immunology of osteosarcoma, immunotherapy is becoming an increasingly attractive treatment strategy. It is generally assumed that adaptive immune cells, especially CTLs, have the greatest potential to eliminate tumors, due to their professional antigen recognition activity and specific killing of tumors (218). However, the characteristics of osteosarcomas (e.g., low expression of MHC-I molecules, absence of specific tumor antigens, and impaired antigen presentation) impede the anti-tumor capacity of CTLs (3, 20). Innate immune cells have unique advantages related to eliminating osteosarcoma due to their roles in antigen presentation, antigen-specific T cell priming, and MHC-independent direct cell killing. Efficacy can be further improved by using auxiliary strategies such as epigenetic modification, gene engineering, and mAb therapy. However, existing immunosuppressive mechanisms, especially the immune checkpoints imposed on immune cells, act as major obstacles to efficacy of innate immune cell-based therapy. Considering the role of OVs in induction of innate immune response, it is reasonable to combine innate immune cell-based therapy with ICPIs or OVs to treat osteosarcoma.

## Author Contributions

This review paper was written by ZeW, revised by ZhW, and BL, suggested by SW and TC, and edited and guided by ZY. All authors read and approved the final version of the manuscript.

### Conflict of Interest Statement

The authors declare that the research was conducted in the absence of any commercial or financial relationships that could be construed as a potential conflict of interest. The reviewer BT and handling Editor declared their shared affiliation.

## References

[1] Kansara M Teng MW Smyth MJ Thomas DM. Translational biology of osteosarcoma. Nat Rev Cancer. (2014) 14:722–35. 10.1038/nrc3838 25319867

[2] Isakoff MS Bielack SS Meltzer P Gorlick R. Osteosarcoma: current treatment and a collaborative pathway to success. J Clin Oncol. (2015) 33:3029–35. 10.1200/jco.2014.59.4895 26304877 PMC4979196

[3] Tsukahara T Kawaguchi S Torigoe T Asanuma H Nakazawa E Shimozawa K. Prognostic significance of HLA class I expression in osteosarcoma defined by anti-pan HLA class I monoclonal antibody, EMR8-5. Cancer Sci. (2006) 97:1374–80. 10.1111/j.1349-7006.2006.00317.x 16995877 PMC11158095

[4] Ahmed N Salsman VS Yvon E Louis CU Perlaky L Wels WS. Immunotherapy for osteosarcoma: genetic modification of T cells overcomes low levels of tumor antigen expression. Mol Ther. (2009) 17:1779–87. 10.1038/mt.2009.133 19532139 PMC2835000

[5] Ahmed N Brawley VS Hegde M Robertson C Ghazi A Gerken C. Human epidermal growth factor receptor 2 (HER2) -specific chimeric antigen receptor-modified T cells for the immunotherapy of HER2-positive sarcoma. J Clin Oncol. (2015) 33:1688–96. 10.1200/jco.2014.58.0225 25800760 PMC4429176

[6] Li B Zhu X Sun L Yuan L Zhang J Li H. Induction of a specific CD8+ T-cell response to cancer/testis antigens by demethylating pre-treatment against osteosarcoma. Oncotarget. (2014) 5:10791–802. 10.18632/oncotarget.2505 25301731 PMC4279410

[7] Woo SR Corrales L Gajewski TF. Innate immune recognition of cancer. Ann Rev Immunol. (2015) 33:445–74. 10.1146/annurev-immunol-032414-112043 25622193

[8] Bonneville M Scotet E. Human Vgamma9Vdelta2 T cells: promising new leads for immunotherapy of infections and tumors. Curr Opin Immunol. (2006) 18:539–46. 10.1016/j.coi.2006.07.002 16870417

[9] Tarek N Lee DA. Natural killer cells for osteosarcoma. Adv Exp Med Biol. (2014) 804:341–53. 10.1007/978-3-319-04843-7_19 24924184

[10] Li Z. Potential of human gammadelta T cells for immunotherapy of osteosarcoma. Mol Biol Rep. (2013) 40:427–37. 10.1007/s11033-012-2077-y 23065272

[11] Wang S Li H Ye C Lin P Li B Zhang W. Valproic acid combined with zoledronate enhance gammadelta T cell-mediated cytotoxicity against osteosarcoma cells via the accumulation of mevalonate pathway intermediates. Front Immunol. (2018) 9:377. 10.3389/fimmu.2018.00377 29535738 PMC5835048

[12] Wang Z Wang Z Li S Li B Sun L Li H. Decitabine enhances Vgamma9Vdelta2 T cell-mediated cytotoxic effects on osteosarcoma cells via the NKG2DL-NKG2D axis. Front Immunol. (2018) 9:1239. 10.3389/fimmu.2018.01239 29910819 PMC5992281

[13] Liu M Sun LL Li YJ Li HY Zhang J Li BH. Trastuzumab enhanced the cytotoxicity of Vgamma9Vdelta2 T cells against zoledronate-sensitized osteosarcoma cells. Int Immunopharmacol. (2015) 28:160–7. 10.1016/j.intimp.2015.06.002 26071219

[14] Li Z Peng H Xu Q Ye Z. Sensitization of human osteosarcoma cells to Vgamma9Vdelta2 T-cell-mediated cytotoxicity by zoledronate. J Orthopaedic Res. (2012) 30:824–30. 10.1002/jor.21579 22025284

[15] Tawbi HA Burgess M Bolejack V Van Tine BA Schuetze SM Hu J. Pembrolizumab in advanced soft-tissue sarcoma and bone sarcoma (SARC028): a multicentre, two-cohort, single-arm, open-label, phase 2 trial. Lancet Oncol. (2017) 18:1493–501. 10.1016/s1470-2045(17)30624-1 28988646 PMC7939029

[16] den Haan JM Lehar SM Bevan MJ. CD8(+) but not CD8(-) dendritic cells cross-prime cytotoxic T cells *in vivo*. J Exp Med. (2000) 192:1685–96. 10.1084/jem.192.12.1685 11120766 PMC2213493

[17] Fiore F Castella B Nuschak B Bertieri R Mariani S Bruno B. Enhanced ability of dendritic cells to stimulate innate and adaptive immunity on short-term incubation with zoledronic acid. Blood. (2007) 110:921–7. 10.1182/blood-2006-09-044321 17403919

[18] Van Acker HH Anguille S De Reu H Berneman ZN Smits EL Van Tendeloo VF. Interleukin-15-cultured dendritic cells enhance anti-tumor gamma delta T cell functions through IL-15 secretion. Front Immunol. (2018) 9:658. 10.3389/fimmu.2018.00658 29692776 PMC5902500

[19] Wongkajornsilp A Sangsuriyong S Hongeng S Waikakul S Asavamongkolkul A Huabprasert S. Effective osteosarcoma cytolysis using cytokine-induced killer cells pre-inoculated with tumor RNA-pulsed dendritic cells. J Orthopaedic Res. (2005) 23:1460–6. 10.1016/j.orthres.2005.03.009.1100230632 15908161

[20] Suryawanshi A Manicassamy S. Tumors induce immune tolerance through activation of beta-catenin/TCF4 signaling in dendritic cells: a novel therapeutic target for cancer immunotherapy. Oncoimmunology. (2015) 4:e1052932. 10.1080/2162402x.2015.1052932 26587326 PMC4635893

[21] Cornwall SM Wikstrom M Musk AW Alvarez J Nowak AK Nelson DJ. Human mesothelioma induces defects in dendritic cell numbers and antigen-processing function which predict survival outcomes. Oncoimmunology. (2016) 5:e1082028. 10.1080/2162402x.2015.1082028 27057464 PMC4801471

[22] Yu Z Qian J Wu J Gao J Zhang M. Allogeneic mRNA-based electrotransfection of autologous dendritic cells and specific antitumor effects against osteosarcoma in rats. Med Oncol. (2012) 29:3440–8. 10.1007/s12032-012-0312-y 22843292

[23] Yu Z Sun H Zhang T Yang T Long H Ma B. Specific antitumor effects of tumor vaccine produced by autologous dendritic cells transfected with allogeneic osteosarcoma total RNA through electroporation in rats. Cancer Biol Ther. (2009) 8:973–80. 10.4161/cbt.8.10.8281 19287216

[24] Yu Z Fan Q Hao X Long H. Specific antitumor effects of tumor vaccine produced by electrofusion between osteosarcoma cell and dendritic cell in rats. Cell Mol Immunol. (2004) 1:454–60. 16293215

[25] Yu Z Ma B Zhou Y Zhang M Long H Wang Y. Allogeneic tumor vaccine produced by electrofusion between osteosarcoma cell line and dendritic cells in the induction of antitumor immunity. Cancer Invest. (2007) 25:535–41. 10.1080/07357900701508918 17952746

[26] Yu Z Ma B Zhou Y Zhang M Qiu X Fan Q. Activation of antitumor cytotoxic T lymphocytes by fusion of patient-derived dendritic cells with autologous osteosarcoma. Exp Oncol. (2005) 27:273–8. 16404346

[27] Fang X Jiang C Xia Q. Effectiveness evaluation of dendritic cell immunotherapy for osteosarcoma on survival rate and *in vitro* immune response. Genet Mol Res. (2015) 14:11763–70. 10.4238/2015.October.2.10 26436501

[28] He YT Zhang QM Kou QC Tang B. In vitro generation of cytotoxic T lymphocyte response using dendritic cell immunotherapy in osteosarcoma. Oncol Let. (2016) 12:1101–6. 10.3892/ol.2016.4714 27446401 PMC4950224

[29] Kawano M Nishida H Nakamoto Y Tsumura H Tsuchiya H. Cryoimmunologic antitumor effects enhanced by dendritic cells in osteosarcoma. Clin Orthopaedics Relat Res. (2010) 468:1373–83. 10.1007/s11999-010-1302-z 20232181 PMC2853649

[30] Kawano M Itonaga I Iwasaki T Tsuchiya H Tsumura H. Anti-TGF-beta antibody combined with dendritic cells produce antitumor effects in osteosarcoma. Clin Orthopaedics Relat Res. (2012) 470:2288–94. 10.1007/s11999-012-2299-2 22415727 PMC3392369

[31] Kawano M Itonaga I Iwasaki T Tsumura H. Enhancement of antitumor immunity by combining anti-cytotoxic T lymphocyte antigen-4 antibodies and cryotreated tumor lysate-pulsed dendritic cells in murine osteosarcoma. Oncol Rep. (2013) 29:1001–6. 10.3892/or.2013.2224 23291864

[32] Kawano M Tanaka K Itonaga I Iwasaki T Miyazaki M Ikeda S. Dendritic cells combined with anti-GITR antibody produce antitumor effects in osteosarcoma. Oncol Rep. (2015) 34:1995–2001. 10.3892/or.2015.4161 26239052

[33] Garg AD Vara Perez M Schaaf M Agostinis P Zitvogel L Kroemer G. Trial watch: dendritic cell-based anticancer immunotherapy. Oncoimmunology. (2017) 6:e1328341. 10.1080/2162402x.2017.1328341 28811970 PMC5543823

[34] Krishnadas DK Shusterman S Bai F Diller L Sullivan JE Cheerva AC. A phase I trial combining decitabine/dendritic cell vaccine targeting MAGE-A1, MAGE-A3 and NY-ESO-1 for children with relapsed or therapy-refractory neuroblastoma and sarcoma. Cancer Immunol Immunother. (2015) 64:1251–60. 10.1007/s00262-015-1731-3 26105625 PMC11028635

[35] Himoudi N Wallace R Parsley KL Gilmour K Barrie AU Howe K. Lack of T-cell responses following autologous tumour lysate pulsed dendritic cell vaccination, in patients with relapsed osteosarcoma. Clin Trans Oncol. (2012) 14:271–9. 10.1007/s12094-012-0795-1 22484634

[36] Miwa S Nishida H Tanzawa Y Takeuchi A Hayashi K Yamamoto N. Phase 1/2 study of immunotherapy with dendritic cells pulsed with autologous tumor lysate in patients with refractory bone and soft tissue sarcoma. Cancer. (2017) 123:1576–84. 10.1002/cncr.30606 28241093

[37] Tan Z Zhou J Cheung AK Yu Z Cheung KW Liang J. Vaccine-elicited CD8+ T cells cure mesothelioma by overcoming tumor-induced immunosuppressive environment. Cancer Res. (2014) 74:6010–21. 10.1158/0008-5472.can-14-0473 25125656

[38] Noy R Pollard JW. Tumor-associated macrophages: from mechanisms to therapy. Immunity. (2014) 41:49–61. 10.1016/j.immuni.2014.06.010 25035953 PMC4137410

[39] Segaliny AI Mohamadi A Dizier B Lokajczyk A Brion R Lanel R. Interleukin-34 promotes tumor progression and metastatic process in osteosarcoma through induction of angiogenesis and macrophage recruitment. Int J Cancer. (2015) 137:73–85. 10.1002/ijc.29376 25471534

[40] Han Y Guo W Ren T Huang Y Wang S Liu K. Tumor-associated macrophages promote lung metastasis and induce epithelial-mesenchymal transition in osteosarcoma by activating the COX-2/STAT3 axis. Cancer Lett. (2019) 440–441:116–25. 10.1016/j.canlet.2018.10.011 30343113

[41] Han Q Shi H Liu F. CD163(+) M2-type tumor-associated macrophage support the suppression of tumor-infiltrating T cells in osteosarcoma. Int Immunopharmacol. (2016) 34:101–6. 10.1016/j.intimp.2016.01.023 26938675

[42] Li X Chen Y Liu X Zhang J He X Teng G. Tim3/Gal9 interactions between T cells and monocytes result in an immunosuppressive feedback loop that inhibits Th1 responses in osteosarcoma patients. Int Immunopharmacol. (2017) 44:153–9. 10.1016/j.intimp.2017.01.006 28103502

[43] Buddingh EP Kuijjer ML Duim RA Burger H Agelopoulos K Myklebost O. Tumor-infiltrating macrophages are associated with metastasis suppression in high-grade osteosarcoma: a rationale for treatment with macrophage activating agents. Clin Cancer Res. (2011) 17:2110–9. 10.1158/1078-0432.ccr-10-2047 21372215

[44] Ratti C Botti L Cancila V Galvan S Torselli I Garofalo C. Trabectedin overrides osteosarcoma differentiative block and reprograms the tumor immune environment enabling effective combination with immune checkpoint inhibitors. Clin Cancer Res. (2017) 23:5149–61. 10.1158/1078-0432.ccr-16-3186 28600479

[45] Gomez-Brouchet A Illac C Gilhodes J Bouvier C Aubert S Guinebretiere JM. CD163-positive tumor-associated macrophages and CD8-positive cytotoxic lymphocytes are powerful diagnostic markers for the therapeutic stratification of osteosarcoma patients: an immunohistochemical analysis of the biopsies fromthe French OS2006 phase 3 trial. Oncoimmunology. (2017) 6:e1331193. 10.1080/2162402x.2017.1331193 28932633 PMC5599091

[46] Zhou Q Xian M Xiang S Xiang D Shao X Wang J. All-trans retinoic acid prevents osteosarcoma metastasis by inhibiting M2 polarization of tumor-associated macrophages. Cancer Immunol Res. (2017) 5:547–59. 10.1158/2326-6066.cir-16-0259 28515123

[47] Kimura Y Sumiyoshi M. Resveratrol prevents tumor growth and metastasis by inhibiting lymphangiogenesis and M2 macrophage activation and differentiation in tumor-associated macrophages. Nutr Cancer. (2016) 68:667–78. 10.1080/01635581.2016.1158295 27145432

[48] Kimura Y Sumiyoshi M Antitumor and antimetastatic actions of dihydroxycoumarins (esculetin or fraxetin) through the inhibition of M2 macrophage differentiation in tumor-associated macrophages and/or G1 arrest in tumor cells. Eur J Pharmacol. (2015) 746:115–25. 10.1016/j.ejphar.2014.10.048 25445053

[49] Zhao SJ Jiang YQ Xu NW Li Q Zhang Q Wang SY. SPARCL1 suppresses osteosarcoma metastasis and recruits macrophages by activation of canonical WNT/beta-catenin signaling through stabilization of the WNT-receptor complex. Oncogene. (2018) 37:1049–61. 10.1038/onc.2017.403 29084211 PMC5851113

[50] Schroit AJ Fidler IJ. Effects of liposome structure and lipid composition on the activation of the tumoricidal properties of macrophages by liposomes containing muramyl dipeptide. Cancer Res. (1982) 42:161–7. 7053846

[51] Pahl J Kwappenberg K Varypataki E Santos S Kuijjer M Mohamed S. Macrophages inhibit human osteosarcoma cell growth after activation with the bacterial cell wall derivative liposomal muramyl tripeptide in combination with interferon-γ. J Exp Clin Cancer Res. (2014) 33:27. 10.1186/1756-9966-33-27 24612598 PMC4007518

[52] Meyers PA Schwartz CL Krailo MD Healey JH Bernstein ML Betcher D. Osteosarcoma: the addition of muramyl tripeptide to chemotherapy improves overall survival–a report from the Children's Oncology Group. J Clin Oncol. (2008) 26:633–8. 10.1200/jco.2008.14.0095 18235123

[53] Chou AJ Kleinerman ES Krailo MD Chen Z Betcher DL Healey JH. Addition of muramyl tripeptide to chemotherapy for patients with newly diagnosed metastatic osteosarcoma: a report from the Children's Oncology Group. Cancer. (2009) 115:5339–48. 10.1002/cncr.24566 19637348 PMC2783515

[54] Spits H Artis D Colonna M Diefenbach A Di Santo JP Eberl G. Innate lymphoid cells–a proposal for uniform nomenclature. Nat Rev Immunol. (2013) 13:145–9. 10.1038/nri3365 23348417

[55] Bottcher JP Bonavita E Chakravarty P Blees H Cabeza-Cabrerizo M Sammicheli S. NK cells stimulate recruitment of cDC1 into the tumor microenvironment promoting cancer immune control. Cell. (2018) 172:1022–37.e1014. 10.1016/j.cell.2018.01.004 29429633 PMC5847168

[56] Markiewicz K Zeman K Kozar A Golebiowska-Wawrzyniak M Wozniak W. Evaluation of selected parameters of cellular immunity in children with osteosarcoma at diagnosis. Medycyna wieku rozwojowego. (2012) 16:212–21. 23378399

[57] Luksch R Perotti D Cefalo G Gambacorti Passerini C Massimino M Spreafico F. Immunomodulation in a treatment program including pre- and post-operative interleukin-2 and chemotherapy for childhood osteosarcoma. Tumori. (2003) 89:263–8. 10.1177/030089160308900306 12908780

[58] Mariani E Tarozzi A Meneghetti A Cattini L Facchini A. Human osteosarcoma cell susceptibility to natural killer cell lysis depends on CD54 and increases after TNF alpha incubation. FEBS Lett. (1997) 406:83–8. 9109391 10.1016/s0014-5793(97)00247-0

[59] Meneghetti A Mariani E Santi S Riccio M Cattini L Paoletti S. NK binding capacity and lytic activity depend on the expression of ICAM-1 on target bone tumours. Int J Oncol. (1999) 15:909–14. 10536172 10.3892/ijo.15.5.909

[60] Buddingh EP Schilham MW Ruslan SE Berghuis D Szuhai K Suurmond J. Chemotherapy-resistant osteosarcoma is highly susceptible to IL-15-activated allogeneic and autologous NK cells. Cancer Immunol Immunother. (2011) 60:575–86. 10.1007/s00262-010-0965-3 21240486 PMC3061210

[61] Lu SM Xiao P Xue L Che LH Yang P Li Y. Prevalent expression of MHC class I chain-related molecule A in human osteosarcoma. Neoplasma. (2008) 55:266–72. 18348660

[62] Yamanegi K Yamane J Kobayashi K Kato-Kogoe N Ohyama H Nakasho K. Sodium valproate, a histone deacetylase inhibitor, augments the expression of cell-surface NKG2D ligands, MICA/B, without increasing their soluble forms to enhance susceptibility of human osteosarcoma cells to NK cell-mediated cytotoxicity. Oncol Rep. (2010) 24:1621–7. 10.3892/or_00001026 21042760

[63] Kiany S Huang G Kleinerman ES. Effect of entinostat on NK cell-mediated cytotoxicity against osteosarcoma cells and osteosarcoma lung metastasis. Oncoimmunology. (2017) 6:e1333214. 10.1080/2162402x.2017.1333214 28919994 PMC5593704

[64] Rao-Bindal K Zhou Z Kleinerman ES. MS-275 sensitizes osteosarcoma cells to Fas ligand-induced cell death by increasing the localization of Fas in membrane lipid rafts. Cell Death Dis. (2012) 3:e369. 10.1038/cddis.2012.101 22875006 PMC3434650

[65] Yamanegi K Yamane J Kobayashi K Kato-Kogoe N Ohyama H Nakasho K. Valproic acid cooperates with hydralazine to augment the susceptibility of human osteosarcoma cells to Fas- and NK cell-mediated cell death. Int J Oncol. (2012) 41:83–91. 10.3892/ijo.2012.1438 22576685

[66] Pahl JH Ruslan SE Kwappenberg KM van Ostaijen-Ten Dam MM van Tol MJ Lankester AC. Antibody-dependent cell lysis by NK cells is preserved after sarcoma-induced inhibition of NK cell cytotoxicity. Cancer Immunol Immunother. (2013) 62:1235–47. 10.1007/s00262-013-1406-x 23624801 PMC11028949

[67] Liebau C Merk H Schmidt S Roesel C Karreman C Prisack JB. Interleukin-12 and interleukin-18 change ICAM-I expression, and enhance natural killer cell mediated cytolysis of human osteosarcoma cells. Cytokines Cell Mol Ther. (2002) 7:135–42. 10.1080/13684730310001977 14660053

[68] Mariani E Meneghetti A Tarozzi A Cattini L Facchini A. Interleukin-12 induces efficient lysis of natural killer-sensitive and natural killer-resistant human osteosarcoma cells: the synergistic effect of interleukin-2. Scand J Immunol. (2000) 51:618–25. 10.1046/j.1365-3083.2000.00737.x 10849374

[69] Honorati MC Neri S Cattini L Facchini A. IL-17 enhances the susceptibility of U-2 OS osteosarcoma cells to NK cell lysis. Clin Exp Immunol. (2003) 133:344–9. 10.1046/j.1365-2249.2003.02234.x 12930359 PMC1808781

[70] Pahl JH Ruslan SE Buddingh EP Santos SJ Szuhai K Serra M. Anti-EGFR antibody cetuximab enhances the cytolytic activity of natural killer cells toward osteosarcoma. Clin Cancer Res. (2012) 18:432–41. 10.1158/1078-0432.ccr-11-2277 22090361

[71] Zingoni A Fionda C Borrelli C Cippitelli M Santoni A Soriani A. Natural killer cell response to chemotherapy-stressed cancer cells: role in tumor immunosurveillance. Front Immunol. (2017) 8:1194. 10.3389/fimmu.2017.01194 28993779 PMC5622151

[72] Gordon N Koshkina NV Jia SF Khanna C Mendoza A Worth LL. Corruption of the Fas pathway delays the pulmonary clearance of murine osteosarcoma cells, enhances their metastatic potential, and reduces the effect of aerosol gemcitabine. Clin Cancer Res. (2007) 13:4503–10. 10.1158/1078-0432.ccr-07-0313 17671136 PMC4503209

[73] Henney CS Kuribayashi K Kern DE Gillis S. Interleukin-2 augments natural killer cell activity. Nature. (1981) 291:335–8. 6164929 10.1038/291335a0

[74] Nguyen R Houston J Chan WK Finkelstein D Dyer MA. The role of interleukin-2, all-trans retinoic acid, and natural killer cells: surveillance mechanisms in anti-GD2 antibody therapy in neuroblastoma. Cancer Immunol Immunother. (2018) 67:615–26. 10.1007/s00262-017-2108-6 29327110 PMC5862751

[75] Ladenstein R Potschger U Valteau-Couanet D Luksch R Castel V Yaniv I. Interleukin 2 with anti-GD2 antibody ch14.18/CHO (dinutuximab beta) in patients with high-risk neuroblastoma (HR-NBL1/SIOPEN): a multicentre, randomised, phase 3 trial. Lancet Oncol. (2018) 19:1617–29. 10.1016/s1470-2045(18)30578-3 30442501

[76] Guma SR Lee DA Ling Y Gordon N Kleinerman ES. Aerosol interleukin-2 induces natural killer cell proliferation in the lung and combination therapy improves the survival of mice with osteosarcoma lung metastasis. Pediatr Blood Cancer. (2014) 61:1362–8. 10.1002/pbc.25019 24610870 PMC4144337

[77] Khanna C Anderson PM Hasz DE Katsanis E Neville M Klausner JS. Interleukin-2 liposome inhalation therapy is safe and effective for dogs with spontaneous pulmonary metastases. Cancer. (1997) 79:1409–21. 9083164 10.1002/(sici)1097-0142(19970401)79:7<1409::aid-cncr19>3.0.co;2-3

[78] Ruggeri L Urbani E Andre P Mancusi A Tosti A Topini F. Effects of anti-NKG2A antibody administration on leukemia and normal hematopoietic cells. Haematologica. (2016) 101:626–33. 10.3324/haematol.2015.135301 26721894 PMC5004363

[79] Vey N Bourhis JH Boissel N Bordessoule D Prebet T Charbonnier A. A phase 1 trial of the anti-inhibitory KIR mAb IPH2101 for AML in complete remission. Blood. (2012) 120:4317–23. 10.1182/blood-2012-06-437558 23002117

[80] Chang YH Connolly J Shimasaki N Mimura K Kono K Campana D. A chimeric receptor with NKG2D specificity enhances natural killer cell activation and killing of tumor cells. Cancer Res. (2013) 73:1777–86. 10.1158/0008-5472.can-12-3558 23302231

[81] Klingemann H Boissel L Toneguzzo F. Natural killer cells for immunotherapy - advantages of the NK-92 cell line over blood NK cells. Front Immunol. (2016) 7:91. 10.3389/fimmu.2016.00091 27014270 PMC4789404

[82] Zhang C Burger MC Jennewein L Genssler S Schonfeld K Zeiner P. ErbB2/HER2-specific NK cells for targeted therapy of glioblastoma. J Natl Cancer Institute. (2016) 108:375. 10.1093/jnci/djv375 26640245

[83] Suck G Odendahl M Nowakowska P Seidl C Wels WS Klingemann HG. NK-92: an 'off-the-shelf therapeutic' for adoptive natural killer cell-based cancer immunotherapy. Cancer Immunol Immunother. (2016) 65:485–92. 10.1007/s00262-015-1761-x 26559813 PMC11029582

[84] Miller JS Soignier Y Panoskaltsis-Mortari A McNearney SA Yun GH Fautsch SK. Successful adoptive transfer and *in vivo* expansion of human haploidentical NK cells in patients with cancer. Blood. (2005) 105:3051–7. 10.1182/blood-2004-07-2974 15632206

[85] Hermanson DL Bendzick L Pribyl L McCullar V Vogel RI Miller JS. Induced pluripotent stem cell-derived natural killer cells for treatment of ovarian cancer. Stem Cells. (2016) 34:93–101. 10.1002/stem.2230 26503833 PMC4713309

[86] Knorr DA Ni Z Hermanson D Hexum MK Bendzick L Cooper LJ. Clinical-scale derivation of natural killer cells from human pluripotent stem cells for cancer therapy. Stem Cells Trans Med. (2013) 2:274–83. 10.5966/sctm.2012-0084 23515118 PMC3659832

[87] Li Y Hermanson DL Moriarity BS Kaufman DS. Human iPSC-derived natural killer cells engineered with chimeric antigen receptors enhance anti-tumor activity. Cell Stem Cell. (2018) 23:181–92.e185. 10.1016/j.stem.2018.06.002 30082067 PMC6084450

[88] Salas-Mckee J Kong W Gladney WL Jadlowsky JK Plesa G Davis MM CRISPR/Cas9-based genome editing in the era of CAR T cell immunotherapy. Hum Vaccines Immunother. (2019) 93:1–7. 10.1080/21645515.2019.1571893 PMC6605860 30735463

[89] Hu W Zi Z Jin Y Li G Shao K Cai Q. CRISPR/Cas9-mediated PD-1 disruption enhances human mesothelin-targeted CAR T cell effector functions. Cancer Immunol Immunother. (2019) 68:365–77. 10.1007/s00262-018-2281-2 30523370 PMC11028344

[90] Ren J Liu X Fang C Jiang S June CH Zhao Y. Multiplex genome editing to generate universal CAR T cells resistant to PD1 inhibition. Clin Cancer Res. (2017) 23:2255–66. 10.1158/1078-0432.ccr-16-1300 27815355 PMC5413401

[91] Eyquem J Mansilla-Soto J Giavridis T van der Stegen SJ Hamieh M Cunanan KM. Targeting a CAR to the TRAC locus with CRISPR/Cas9 enhances tumour rejection. Nature. (2017) 543:113–7. 10.1038/nature21405 28225754 PMC5558614

[92] Crowe NY Uldrich AP Kyparissoudis K Hammond KJ Hayakawa Y Sidobre S. Glycolipid antigen drives rapid expansion and sustained cytokine production by NK T cells. J Immunol. (2003) 171:4020–7. 10.4049/jimmunol.171.8.4020 14530322

[93] Metelitsa LS Weinberg KI Emanuel PD Seeger RC. Expression of CD1d by myelomonocytic leukemias provides a target for cytotoxic NKT cells. Leukemia. (2003) 17:1068–77. 10.1038/sj.leu.2402943 12764370

[94] Tachibana T Onodera H Tsuruyama T Mori A Nagayama S Hiai H. Increased intratumor Valpha24-positive natural killer T cells: a prognostic factor for primary colorectal carcinomas. Clin Cancer Res. (2005) 11:7322–7. 10.1158/1078-0432.ccr-05-0877 16243803

[95] Hishiki T Mise N Harada K Ihara F Takami M Saito T. Invariant natural killer T infiltration in neuroblastoma with favorable outcome. Pediatr Surg Int. (2018) 34:195–201. 10.1007/s00383-017-4189-x 29018959

[96] Bricard G Cesson V Devevre E Bouzourene H Barbey C Rufer N. Enrichment of human CD4+ V(alpha)24/Vbeta11 invariant NKT cells in intrahepatic malignant tumors. J Immunol. (2009) 182:5140–51. 10.4049/jimmunol.0711086 19342695

[97] Kenna T Golden-Mason L Porcelli SA Koezuka Y Hegarty JE O'Farrelly C. NKT cells from normal and tumor-bearing human livers are phenotypically and functionally distinct from murine NKT cells. J Immunol. (2003) 171:1775–9. 10.4049/jimmunol.171.4.1775 12902477

[98] Li XF Dai D Song XY Liu JJ Zhu L Zhu X. A different representation of natural T cells and natural killer cells between tumor-infiltrating and periphery lymphocytes in human hepatocellular carcinoma. Oncol Lett. (2017) 13:3291–8. 10.3892/ol.2017.5808 28529568 PMC5431491

[99] Fallarini S Paoletti T Orsi Battaglini N Lombardi G. Invariant NKT cells increase drug-induced osteosarcoma cell death. Br J Pharmacol. (2012) 167:1533–49. 10.1111/j.1476-5381.2012.02108.x 22817659 PMC3514765

[100] Terabe M Khanna C Bose S Melchionda F Mendoza A Mackall CL CD1d-restricted natural killer T cells can down-regulate tumor immunosurveillance independent of interleukin-4 receptor-signal transducer and activator of transcription 6 or transforming growth factor-beta. Cancer Res. (2006) 66:3869–75. 10.1158/0008-5472.can-05-3421 16585215

[101] Krijgsman D Hokland M Kuppen PJK. The role of natural killer T cells in cancer-A phenotypical and functional approach. Front Immunol. (2018) 9:367. 10.3389/fimmu.2018.00367 29535734 PMC5835336

[102] Ishikawa A Motohashi S Ishikawa E Fuchida H Higashino K Otsuji M. A phase I study of alpha-galactosylceramide (KRN7000)-pulsed dendritic cells in patients with advanced and recurrent non-small cell lung cancer. Clin Cancer Res. (2005) 11:1910–7. 10.1158/1078-0432.ccr-04-1453 15756017

[103] Motohashi S Nagato K Kunii N Yamamoto H Yamasaki K Okita K. A phase I-II study of alpha-galactosylceramide-pulsed IL-2/GM-CSF-cultured peripheral blood mononuclear cells in patients with advanced and recurrent non-small cell lung cancer. J Immunol. (2009) 182:2492–501. 10.4049/jimmunol.0800126 19201905

[104] Watarai H Fujii S Yamada D Rybouchkin A Sakata S Nagata Y. Murine induced pluripotent stem cells can be derived from and differentiate into natural killer T cells. J Clin Invest. (2010) 120:2610–8. 10.1172/jci42027 20516640 PMC2898602

[105] Yamada D Iyoda T Vizcardo R Shimizu K Sato Y Endo TA. Efficient regeneration of human Valpha24(+) invariant natural killer T cells and their anti-tumor activity *in vivo*. Stem Cells. (2016) 34:2852–60. 10.1002/stem.2465 27422351

[106] Tahir SM Cheng O Shaulov A Koezuka Y Bubley GJ Wilson SB. Loss of IFN-gamma production by invariant NK T cells in advanced cancer. J Immunol. (2001) 167:4046–50. 10.4049/jimmunol.167.7.4046 11564825

[107] Laurent X Bertin B Renault N Farce A Speca S Milhomme O. Switching invariant natural killer T (iNKT) cell response from anticancerous to anti-inflammatory effect: molecular bases. J Med Chem. (2014) 57:5489–508. 10.1021/jm4010863 24428717

[108] Girardi M Oppenheim DE Steele CR Lewis JM Glusac E Filler R. Regulation of cutaneous malignancy by gammadelta T cells. Science. (2001) 294:605–9. 10.1126/science.1063916 11567106

[109] Paul S Lal G. Regulatory and effector functions of gamma-delta (gammadelta) T cells and their therapeutic potential in adoptive cellular therapy for cancer. International journal of cancer. (2016) 139:976-985. 10.1002/ijc.30109 27012367

[110] Brandes M Willimann K Moser B. Professional antigen-presentation function by human gammadelta T Cells. Science. (2005) 309:264–8. 10.1126/science.1110267 15933162

[111] Brandes M Willimann K Bioley G Levy N Eberl M Luo M. Cross-presenting human gammadelta T cells induce robust CD8+ alphabeta T cell responses. Proc Natl Acad Sci USA. (2009) 106:2307–12. 10.1073/pnas.0810059106 19171897 PMC2650152

[112] Altvater B Pscherer S Landmeier S Kailayangiri S Savoldo B Juergens H. Activated human gammadelta T cells induce peptide-specific CD8+ T-cell responses to tumor-associated self-antigens. Cancer Immunol Immunother. (2012) 61:385–96. 10.1007/s00262-011-1111-6 21928126 PMC11028957

[113] Lozupone F Pende D Burgio VL Castelli C Spada M Venditti M. Effect of human natural killer and gammadelta T cells on the growth of human autologous melanoma xenografts in SCID mice. Cancer Res. (2004) 64:378–85. 10.1158/0008-5472.CAN-03-1501 14729648

[114] Yuasa T Sato K Ashihara E Takeuchi M Maita S Tsuchiya N. Intravesical administration of gammadelta T cells successfully prevents the growth of bladder cancer in the murine model. Cancer Immunol Immunother. (2009) 58:493–502. 10.1007/s00262-008-0571-9 18682944 PMC11029835

[115] Fournie JJ Sicard H Poupot M Bezombes C Blanc A Romagne F. What lessons can be learned from gammadelta T cell-based cancer immunotherapy trials? Cell Mol Immunol. (2013) 10:35–41. 10.1038/cmi.2012.39 23241899 PMC4003170

[116] Kato Y Tanaka Y Miyagawa F Yamashita S Minato N. Targeting of tumor cells for human gammadelta T cells by nonpeptide antigens. J Immunol. (2001) 167:5092–8. 10.4049/jimmunol.167.9.5092 11673519

[117] Muraro M Mereuta OM Carraro F Madon E Fagioli F. Osteosarcoma cell line growth inhibition by zoledronate-stimulated effector cells. Cell Immunol. (2007) 249:63–72. 10.1016/j.cellimm.2007.11.005 18163982

[118] Li Z Xu Q Peng H Cheng R Sun Z Ye Z. IFN-gamma enhances HOS and U2OS cell lines susceptibility to gammadelta T cell-mediated killing through the Fas/Fas ligand pathway. Int Immunopharmacol. (2011) 11:496–503. 10.1016/j.intimp.2011.01.001 21238618

[119] Li Z Zhang J Tang J Wang R. Celastrol increases osteosarcoma cell lysis by gammadelta T cells through up-regulation of death receptors. Oncotarget. (2016) 7:84388–97. 10.18632/oncotarget.12756 27768597 PMC5356667

[120] Rosen LS Gordon D Tchekmedyian S Yanagihara R Hirsh V Krzakowski M. Zoledronic acid versus placebo in the treatment of skeletal metastases in patients with lung cancer and other solid tumors: a phase III, double-blind, randomized trial–the Zoledronic Acid Lung Cancer and Other Solid Tumors Study Group. J Clin Oncol. (2003) 21:3150–7. 10.1200/jco.2003.04.105 12915606

[121] Dass CR Choong PF. Zoledronic acid inhibits osteosarcoma growth in an orthotopic model. Mol Cancer Ther. (2007) 6:3263–70. 10.1158/1535-7163.mct-07-0546 18089720

[122] Chen T Berenson J Vescio R Swift R Gilchick A Goodin S. Pharmacokinetics and pharmacodynamics of zoledronic acid in cancer patients with bone metastases. J Clin Pharmacol. (2002) 42:1228–36. 12412821 10.1177/009127002762491316

[123] Scotlandi K Manara MC Hattinger CM Benini S Perdichizzi S Pasello M. Prognostic and therapeutic relevance of HER2 expression in osteosarcoma and Ewing's sarcoma. Eur J Cancer. (2005) 41:1349–61. 10.1016/j.ejca.2005.03.015 15913990

[124] Ebb D Meyers P Grier H Bernstein M Gorlick R Lipshultz SE. Phase II trial of trastuzumab in combination with cytotoxic chemotherapy for treatment of metastatic osteosarcoma with human epidermal growth factor receptor 2 overexpression: a report from the children's oncology group. J Clin Oncol. (2012) 30:2545–51. 10.1200/jco.2011.37.4546 22665540 PMC3397787

[125] Oberg HH Peipp M Kellner C Sebens S Krause S Petrick D. Novel bispecific antibodies increase gammadelta T-cell cytotoxicity against pancreatic cancer cells. Cancer Res. (2014) 74:1349–60. 10.1158/0008-5472.can-13-0675 24448235

[126] Wang Z Li B Ren Y Ye Z. T-cell-based immunotherapy for osteosarcoma: challenges and opportunities. Front Immunol. (2016) 7:353. 10.3389/fimmu.2016.00353 27683579 PMC5021687

[127] van der Veken LT Coccoris M Swart E Falkenburg JH Schumacher TN Heemskerk MH. Alpha beta T cell receptor transfer to gamma delta T cells generates functional effector cells without mixed TCR dimers *in vivo*. J Immunol. (2009) 182:164–70. 10.4049/jimmunol.182.1.164 19109147

[128] Saito T Hochstenbach F Marusic-Galesic S Kruisbeek AM Brenner M Germain RN. Surface expression of only gamma delta and/or alpha beta T cell receptor heterodimers by cells with four (alpha, beta, gamma, delta) functional receptor chains. J Exp Med. (1988) 168:1003–20. 2971751 10.1084/jem.168.3.1003 PMC2189041

[129] Moser B Eberl M. gammadelta T-APCs: a novel tool for immunotherapy? Cell Mol Life Sci. (2011) 68:2443–52. 10.1007/s00018-011-0706-6 21573785 PMC11114695

[130] Li H Pauza CD. Rapamycin increases the yield and effector function of human gammadelta T cells stimulated *in vitro*. Cancer Immunol Immunother. (2011) 60:361–70. 10.1007/s00262-010-0945-7 21107834 PMC3077899

[131] Dao V Liu Y Pandeswara S Svatek RS Gelfond JA Liu A. Immune-stimulatory effects of rapamycin are mediated by stimulation of antitumor gammadelta T cells. Cancer Res. (2016) 76:5970–82. 10.1158/0008-5472.can-16-0091 27569211 PMC5065775

[132] Cao G Wang Q Li G Meng Z Liu H Tong J. mTOR inhibition potentiates cytotoxicity of Vgamma4 gammadelta T cells via up-regulating NKG2D and TNF-alpha. J Leukocyte Biol. (2016) 100:1181–9. 10.1189/jlb.5A0116-053RR 27256566

[133] Twyman-Saint Victor C Rech AJ Maity A Rengan R Pauken KE Stelekati E. Radiation and dual checkpoint blockade activate non-redundant immune mechanisms in cancer. Nature. (2015) 520:373–7. 10.1038/nature14292 25754329 PMC4401634

[134] Wherry EJ. T cell exhaustion. Nat Immunol. (2011) 12:492–9. 10.1038/ni.2035 21739672

[135] Jan CI Tsai WC Harn HJ Shyu WC Liu MC Lu HM. Predictors of Response to Autologous Dendritic Cell Therapy in Glioblastoma Multiforme. Front Immunol. (2018) 9:727. 10.3389/fimmu.2018.00727 29910795 PMC5992384

[136] Ribas A Wolchok JD. Cancer immunotherapy using checkpoint blockade. Science. (2018) 359:1350–5. 10.1126/science.aar4060 29567705 PMC7391259

[137] Rosenblatt J Glotzbecker B Mills H Vasir B Tzachanis D Levine JD. PD-1 blockade by CT-011, anti-PD-1 antibody, enhances *ex vivo* T-cell responses to autologous dendritic cell/myeloma fusion vaccine. J Immunother. (2011) 34:409–18. 10.1097/CJI.0b013e31821ca6ce 21577144 PMC3142955

[138] Nagaoka K Hosoi A Iino T Morishita Y Matsushita H Kakimi K. Dendritic cell vaccine induces antigen-specific CD8(+) T cells that are metabolically distinct from those of peptide vaccine and is well-combined with PD-1 checkpoint blockade. Oncoimmunology. (2018) 7:e1395124. 10.1080/2162402x.2017.1395124 29399391 PMC5790382

[139] Hsu J Hodgins JJ Marathe M Nicolai CJ Bourgeois-Daigneault MC Trevino TN Contribution of NK cells to immunotherapy mediated by PD-1/PD-L1 blockade. J Clin Invest. (2018) 2018:99317 10.1172/jci99317 PMC6159991 30198904

[140] Beldi-Ferchiou A Lambert M Dogniaux S Vely F Vivier E Olive D. PD-1 mediates functional exhaustion of activated NK cells in patients with Kaposi sarcoma. Oncotarget. (2016) 7:72961–77. 10.18632/oncotarget.12150 27662664 PMC5341956

[141] Iraolagoitia XL Spallanzani RG Torres NI Araya RE Ziblat A Domaica CI. NK cells restrain spontaneous antitumor CD8+ T cell priming through PD-1/PD-L1 interactions with dendritic cells. J Immunol. (2016) 197:953–61. 10.4049/jimmunol.1502291 27342842

[142] Bellucci R Martin A Bommarito D Wang K Hansen SH Freeman GJ. Interferon-gamma-induced activation of JAK1 and JAK2 suppresses tumor cell susceptibility to NK cells through upregulation of PD-L1 expression. Oncoimmunology. (2015) 4:e1008824. 10.1080/2162402x.2015.1008824 26155422 PMC4485824

[143] Guo Y Feng X Jiang Y Shi X Xing X Liu X. PD1 blockade enhances cytotoxicity of in vitro expanded natural killer cells towards myeloma cells. Oncotarget. (2016) 7:48360–74. 10.18632/oncotarget.10235 27356741 PMC5217023

[144] Wang F Li B Wei Y Zhao Y Wang L Zhang P. Tumor-derived exosomes induce PD1(+) macrophage population in human gastric cancer that promotes disease progression. Oncogenesis. (2018) 7:41. 10.1038/s41389-018-0049-3 29799520 PMC5968036

[145] Mattox AK Lee J Westra WH Pierce RH Ghossein R Faquin WC. PD-1 Expression in head and neck squamous cell carcinomas derives primarily from functionally anergic CD4(+) TILs in the presence of PD-L1(+) TAMs. Cancer Res. (2017) 77:6365–74. 10.1158/0008-5472.can-16-3453 28947422 PMC5690870

[146] Dhupkar P Gordon N Stewart J Kleinerman ES. Anti-PD-1 therapy redirects macrophages from an M2 to an M1 phenotype inducing regression of OS lung metastases. Cancer Med. (2018) 7:2654–64. 10.1002/cam4.1518 29733528 PMC6010882

[147] Krempski J Karyampudi L Behrens MD Erskine CL Hartmann L Dong H. Tumor-infiltrating programmed death receptor-1+ dendritic cells mediate immune suppression in ovarian cancer. J Immunol. (2011) 186:6905–13. 10.4049/jimmunol.1100274 21551365 PMC3110549

[148] Shen JK Cote GM Choy E Yang P Harmon D Schwab J. Programmed cell death ligand 1 expression in osteosarcoma. Cancer Immunol Res. (2014) 2:690–8. 10.1158/2326-6066.cir-13-0224 24866169 PMC4082476

[149] Huang X Zhang W Zhang Z Shi D Wu F Zhong B. Prognostic value of programmed cell death 1 ligand-1 (PD-L1) or PD-1 expression in patients with osteosarcoma: a meta-analysis. J Cancer. (2018) 9:2525–31. 10.7150/jca.25011 30026851 PMC6036896

[150] Lussier DM O'Neill L Nieves LM McAfee MS Holechek SA Collins AW. Enhanced T-cell immunity to osteosarcoma through antibody blockade of PD-1/PD-L1 interactions. J Immunother. (2015) 38:96–106. 10.1097/cji.0000000000000065 25751499 PMC6426450

[151] Lussier DM Johnson JL Hingorani P Blattman JN. Combination immunotherapy with alpha-CTLA-4 and alpha-PD-L1 antibody blockade prevents immune escape and leads to complete control of metastatic osteosarcoma. J Immunother Cancer. (2015) 3:21. 10.1186/s40425-015-0067-z 25992292 PMC4437699

[152] Zheng B Ren T Huang Y Sun K Wang S Bao X PD-1 axis expression in musculoskeletal tumors and antitumor effect of nivolumab in osteosarcoma model of humanized mouse. J Hematol Oncol. (2018) 11:16 10.1186/s13045-018-0560-1 29409495 PMC5801803

[153] Galon J Bruni D Approaches to treat immune hot, altered and cold tumours with combination immunotherapies. Nat Rev Drug Discov. (2019) 2019:0007 10.1038/s41573-018-0007-y 30610226

[154] Nuti M Zizzari IG Botticelli A Rughetti A Marchetti P. The ambitious role of anti angiogenesis molecules: Turning a cold tumor into a hot one. Cancer Treat Rev. (2018) 70:41–6. 10.1016/j.ctrv.2018.07.016 30077081

[155] Popovic A Jaffee EM Zaidi N. Emerging strategies for combination checkpoint modulators in cancer immunotherapy. J Clin Invest. (2018) 128:3209–18. 10.1172/jci120775 30067248 PMC6063475

[156] Rudd CE Taylor A Schneider H. CD28 and CTLA-4 coreceptor expression and signal transduction. Immunol Rev. (2009) 229:12–26. 10.1111/j.1600-065X.2009.00770.x 19426212 PMC4186963

[157] Wilgenhof S Corthals J Heirman C van Baren N Lucas S Kvistborg P. Phase II study of autologous monocyte-derived mRNA electroporated dendritic cells (TriMixDC-MEL) plus ipilimumab in patients with pretreated advanced melanoma. J Clin Oncol. (2016) 34:1330–8. 10.1200/jco.2015.63.4121 26926680

[158] Stojanovic A Fiegler N Brunner-Weinzierl M Cerwenka A. CTLA-4 is expressed by activated mouse NK cells and inhibits NK Cell IFN-gamma production in response to mature dendritic cells. J Immunol. (2014) 192:4184–91. 10.4049/jimmunol.1302091 24688023

[159] Liu J Wang J Jiang W Tang Y. Effect of cytotoxic T-lymphocyte antigen-4, TNF-alpha polymorphisms on osteosarcoma: evidences from a meta-analysis. Chin J Cancer Res. (2013) 25:671–8. 10.3978/j.issn.1000-9604.2013.11.06 24385694 PMC3872545

[160] Anderson AC. Tim-3: an emerging target in the cancer immunotherapy landscape. Cancer Immunol Res. (2014) 2:393–8. 10.1158/2326-6066.cir-14-0039 24795351

[161] Sakuishi K Apetoh L Sullivan JM Blazar BR Kuchroo VK Anderson AC. Targeting Tim-3 and PD-1 pathways to reverse T cell exhaustion and restore anti-tumor immunity. J Exp Med. (2010) 207:2187–94. 10.1084/jem.20100643 20819927 PMC2947065

[162] Jin HT Anderson AC Tan WG West EE Ha SJ Araki K. Cooperation of Tim-3 and PD-1 in CD8 T-cell exhaustion during chronic viral infection. Proc Natl Acad Sci USA. (2010) 107:14733–8. 10.1073/pnas.1009731107 20679213 PMC2930455

[163] Das M Zhu C Kuchroo VK. Tim-3 and its role in regulating anti-tumor immunity. Immunol Rev. (2017) 276:97–111. 10.1111/imr.12520 28258697 PMC5512889

[164] da Silva IP Gallois A Jimenez-Baranda S Khan S Anderson AC Kuchroo VK. Reversal of NK-cell exhaustion in advanced melanoma by Tim-3 blockade. Cancer Immunol Res. (2014) 2:410–22. 10.1158/2326-6066.cir-13-0171 24795354 PMC4046278

[165] Gallois A Silva I Osman I Bhardwaj N. Reversal of natural killer cell exhaustion by TIM-3 blockade. Oncoimmunology. (2014) 3:e946365. 10.4161/21624011.2014.946365 25964857 PMC4353130

[166] Xu L Huang Y Tan L Yu W Chen D Lu C. Increased Tim-3 expression in peripheral NK cells predicts a poorer prognosis and Tim-3 blockade improves NK cell-mediated cytotoxicity in human lung adenocarcinoma. International immunopharmacology. (2015) 29:635-641. 10.1016/j.intimp.2015.09.017 26428847

[167] Seo H Jeon I Kim BS Park M Bae EA Song B. IL-21-mediated reversal of NK cell exhaustion facilitates anti-tumour immunity in MHC class I-deficient tumours. Nat Commun. (2017) 8:15776. 10.1038/ncomms15776 28585539 PMC5467212

[168] Frisancho-Kiss S Nyland JF Davis SE Barrett MA Gatewood SJ Njoku DB. Cutting edge: T cell Ig mucin-3 reduces inflammatory heart disease by increasing CTLA-4 during innate immunity. J Immunol. (2006) 176:6411–5. 10.4049/jimmunol.176.11.6411 16709797

[169] Jiang X Yu J Shi Q Xiao Y Wang W Chen G. Tim-3 promotes intestinal homeostasis in DSS colitis by inhibiting M1 polarization of macrophages. Clin Immunol. (2015) 160:328–35. 10.1016/j.clim.2015.07.008 26208474

[170] Yan W Liu X Ma H Zhang H Song X Gao L. Tim-3 fosters HCC development by enhancing TGF-beta-mediated alternative activation of macrophages. Gut. (2015) 64:1593–604. 10.1136/gutjnl-2014-307671 25608525

[171] Maurya N Gujar R Gupta M Yadav V Verma S Sen P. Immunoregulation of dendritic cells by the receptor T cell Ig and mucin protein-3 via Bruton's tyrosine kinase and c-Src. J Immunol. (2014) 193:3417–25. 10.4049/jimmunol.1400395 25172495

[172] Chiba S Baghdadi M Akiba H Yoshiyama H Kinoshita I Dosaka-Akita H. Tumor-infiltrating DCs suppress nucleic acid-mediated innate immune responses through interactions between the receptor TIM-3 and the alarmin HMGB1. Nat Immunol. (2012) 13:832–42. 10.1038/ni.2376 22842346 PMC3622453

[173] Gogoi D Biswas D Borkakoty B Mahanta J Exposure to Plasmodium vivax is associated with the increased expression of exhaustion markers on gammadelta T lymphocytes. Parasite Immunol. (2018) 2018:e12594 10.1111/pim.12594 30276843

[174] Schofield L Ioannidis LJ Karl S Robinson LJ Tan QY Poole DP. Synergistic effect of IL-12 and IL-18 induces TIM3 regulation of gammadelta T cell function and decreases the risk of clinical malaria in children living in Papua New Guinea. BMC Med. (2017) 15:114. 10.1186/s12916-017-0883-8 28615061 PMC5471992

[175] Mumprecht S Schurch C Schwaller J Solenthaler M Ochsenbein AF. Programmed death 1 signaling on chronic myeloid leukemia-specific T cells results in T-cell exhaustion and disease progression. Blood. (2009) 114:1528–36. 10.1182/blood-2008-09-179697 19420358

[176] Ngiow SF von Scheidt B Akiba H Yagita H Teng MW Smyth MJ. Anti-TIM3 antibody promotes T cell IFN-gamma-mediated antitumor immunity and suppresses established tumors. Cancer Res. (2011) 71:3540–51. 10.1158/0008-5472.can-11-0096 21430066

[177] Koyama S Akbay EA Li YY Herter-Sprie GS Buczkowski KA Richards WG. Adaptive resistance to therapeutic PD-1 blockade is associated with upregulation of alternative immune checkpoints. Nat Commun. (2016) 7:10501. 10.1038/ncomms10501 26883990 PMC4757784

[178] Sevigny CP Li L Awad AS Huang L McDuffie M Linden J. Activation of adenosine 2A receptors attenuates allograft rejection and alloantigen recognition. J Immunol. (2007) 178:4240–9. 10.4049/jimmunol.178.7.4240 17371980

[179] Canale FP Ramello MC Nunez N Araujo Furlan CL Bossio SN Gorosito Serran M CD39 expression defines cell exhaustion in tumor-infiltrating CD8(+) T cells. Cancer Res. (2018) 78:115–28. 10.1158/0008-5472.can-16-2684 29066514

[180] Young A Ngiow SF Barkauskas DS Sult E Hay C Blake SJ. Co-inhibition of CD73 and A2AR adenosine signaling improves anti-tumor immune responses. Cancer Cell. (2016) 30:391–403. 10.1016/j.ccell.2016.06.025 27622332

[181] Stagg J Divisekera U Duret H Sparwasser T Teng MW Darcy PK. CD73-deficient mice have increased antitumor immunity and are resistant to experimental metastasis. Cancer Res. (2011) 71:2892–900. 10.1158/0008-5472.can-10-4246 21292811

[182] Stagg J Beavis PA Divisekera U Liu MC Moller A Darcy PK. CD73-deficient mice are resistant to carcinogenesis. Cancer Res. (2012) 72:2190–6. 10.1158/0008-5472.can-12-0420 22396496

[183] Ohta A Gorelik E Prasad SJ Ronchese F Lukashev D Wong MK. A2A adenosine receptor protects tumors from antitumor T cells. Proc Natl Acad Sci USA. (2006) 103:13132–7. 10.1073/pnas.0605251103 16916931 PMC1559765

[184] Wang L Fan J Thompson LF Zhang Y Shin T Curiel TJ. CD73 has distinct roles in nonhematopoietic and hematopoietic cells to promote tumor growth in mice. J Clin Invest. (2011) 121:2371–82. 10.1172/jci45559 21537079 PMC3104756

[185] Jadidi-Niaragh F Atyabi F Rastegari A Kheshtchin N Arab S Hassannia H. CD73 specific siRNA loaded chitosan lactate nanoparticles potentiate the antitumor effect of a dendritic cell vaccine in 4T1 breast cancer bearing mice. J Controll Release. (2017) 246:46–59. 10.1016/j.jconrel.2016.12.012 27993599

[186] Arab S Kheshtchin N Ajami M Ashurpoor M Safvati A Namdar A. Increased efficacy of a dendritic cell-based therapeutic cancer vaccine with adenosine receptor antagonist and CD73 inhibitor. Tumour Biol. (2017) 39:1010428317695021. 10.1177/1010428317695021 28349824

[187] Beavis PA Divisekera U Paget C Chow MT John LB Devaud C. Blockade of A2A receptors potently suppresses the metastasis of CD73+ tumors. Proc Natl Acad Sci USA. (2013) 110:14711–6. 10.1073/pnas.1308209110 23964122 PMC3767556

[188] Raskovalova T Huang X Sitkovsky M Zacharia LC Jackson EK Gorelik E. Gs protein-coupled adenosine receptor signaling and lytic function of activated NK cells. J Immunol. (2005) 175:4383–91. 10.4049/jimmunol.175.7.4383 16177079

[189] Lokshin A Raskovalova T Huang X Zacharia LC Jackson EK Gorelik E. Adenosine-mediated inhibition of the cytotoxic activity and cytokine production by activated natural killer cells. Cancer Res. (2006) 66:7758–65. 10.1158/0008-5472.can-06-0478 16885379

[190] Mittal D Young A Stannard K Yong M Teng MW Allard B. Antimetastatic effects of blocking PD-1 and the adenosine A2A receptor. Cancer Res. (2014) 74:3652–8. 10.1158/0008-5472.can-14-0957 24986517

[191] Young A Ngiow SF Gao Y Patch AM Barkauskas DS Messaoudene M. A2AR Adenosine signaling suppresses natural killer cell maturation in the tumor microenvironment. Cancer Res. (2018) 78:1003–16. 10.1158/0008-5472.can-17-2826 29229601

[192] Novitskiy SV Ryzhov S Zaynagetdinov R Goldstein AE Huang Y Tikhomirov OY. Adenosine receptors in regulation of dendritic cell differentiation and function. Blood. (2008) 112:1822–31. 10.1182/blood-2008-02-136325 18559975 PMC2518889

[193] Panther E Corinti S Idzko M Herouy Y Napp M la Sala A. Adenosine affects expression of membrane molecules, cytokine and chemokine release, and the T-cell stimulatory capacity of human dendritic cells. Blood. (2003) 101:3985–90. 10.1182/blood-2002-07-2113 12446452

[194] Cekic C Sag D Li Y Theodorescu D Strieter RM Linden J. Adenosine A2B receptor blockade slows growth of bladder and breast tumors. J Immunol. (2012) 188:198–205. 10.4049/jimmunol.1101845 22116822 PMC3819109

[195] Li L Hao JX Fredholm BB Schulte G Wiesenfeld-Hallin Z Xu XJ. Peripheral adenosine A2A receptors are involved in carrageenan-induced mechanical hyperalgesia in mice. Neuroscience. (2010) 170:923–8. 10.1016/j.neuroscience.2010.07.045 20678550

[196] Hasko G Pacher P. Regulation of macrophage function by adenosine. Arterioscler Thrombosis Vasc Biol. (2012) 32:865–9. 10.1161/atvbaha.111.226852 22423038 PMC3387535

[197] d'Almeida SM Kauffenstein G Roy C Basset L Papargyris L Henrion D. The ecto-ATPDase CD39 is involved in the acquisition of the immunoregulatory phenotype by M-CSF-macrophages and ovarian cancer tumor-associated macrophages: regulatory role of IL-27. Oncoimmunology. (2016) 5:e1178025. 10.1080/2162402x.2016.1178025 27622030 PMC5006905

[198] Montalban Del Barrio I Penski C Schlahsa L Stein RG Diessner J Wockel A. Adenosine-generating ovarian cancer cells attract myeloid cells which differentiate into adenosine-generating tumor associated macrophages - a self-amplifying, CD39- and CD73-dependent mechanism for tumor immune escape. J Immunother Cancer. (2016) 4:49. 10.1186/s40425-016-0154-9 27532024 PMC4986205

[199] Gruenbacher G Gander H Rahm A Idzko M Nussbaumer O Thurnher M. Ecto-ATPase CD39 inactivates isoprenoid-derived Vgamma9Vdelta2 T cell phosphoantigens. Cell Rep. (2016) 16:444–56. 10.1016/j.celrep.2016.06.009 27346340

[200] Nowak M Lynch L Yue S Ohta A Sitkovsky M Balk SP. The A2aR adenosine receptor controls cytokine production in iNKT cells. Eur J Immunol. (2010) 40:682–7. 10.1002/eji.200939897 20039304 PMC2967447

[201] Semenza GL. Hypoxia-inducible factors in physiology and medicine. Cell. (2012) 148:399–408. 10.1016/j.cell.2012.01.021 22304911 PMC3437543

[202] Synnestvedt K Furuta GT Comerford KM Louis N Karhausen J Eltzschig HK. Ecto-5'-nucleotidase (CD73) regulation by hypoxia-inducible factor-1 mediates permeability changes in intestinal epithelia. J Clin Invest. (2002) 110:993–1002. 10.1172/jci15337 12370277 PMC151145

[203] Guan G Zhang Y Lu Y Liu L Shi D Wen Y. The HIF-1alpha/CXCR4 pathway supports hypoxia-induced metastasis of human osteosarcoma cells. Cancer Lett. (2015) 357:254–64. 10.1016/j.canlet.2014.11.034 25444927

[204] Tonn T Schwabe D Klingemann HG Becker S Esser R Koehl U. Treatment of patients with advanced cancer with the natural killer cell line NK-92. Cytotherapy. (2013) 15:1563–70. 10.1016/j.jcyt.2013.06.017 24094496

[205] Gujar S Bell J Diallo JS. SnapShot: cancer immunotherapy with oncolytic viruses. Cell. (2019) 176:1240-1240.e1241. 10.1016/j.cell.2019.01.051 30794777

[206] Bhat R Dempe S Dinsart C Rommelaere J. Enhancement of NK cell antitumor responses using an oncolytic parvovirus. Int J Cancer. (2011) 128:908–19. 10.1002/ijc.25415 20473905

[207] Ye JF Qi WX Liu MY Li Y. The combination of NK and CD8+T cells with CCL20/IL15-armed oncolytic adenoviruses enhances the growth suppression of TERT-positive tumor cells. Cell Immunol. (2017) 318:35–41. 10.1016/j.cellimm.2017.06.002 28651743

[208] Yoo JY Jaime-Ramirez AC Bolyard C Dai H Nallanagulagari T Wojton J. Bortezomib treatment sensitizes oncolytic HSV-1-treated tumors to NK cell immunotherapy. Clin Cancer Res. (2016) 22:5265–76. 10.1158/1078-0432.ccr-16-1003 27390350 PMC5093037

[209] Benencia F Courreges MC Conejo-Garcia JR Mohamed-Hadley A Zhang L Buckanovich RJ. HSV oncolytic therapy upregulates interferon-inducible chemokines and recruits immune effector cells in ovarian cancer. Mol Ther. (2005) 12:789–802. 10.1016/j.ymthe.2005.03.026 15925544

[210] Meisen WH Wohleb ES Jaime-Ramirez AC Bolyard C Yoo JY Russell L. The impact of macrophage- and microglia-secreted TNFalpha on oncolytic HSV-1 therapy in the glioblastoma tumor microenvironment. Clin Cancer Res. (2015) 21:3274–85. 10.1158/1078-0432.ccr-14-3118 25829396 PMC4780415

[211] Russell SJ Peng KW Bell JC. Oncolytic virotherapy. Nat Biotechnol. (2012) 30:658–70. 10.1038/nbt.2287 22781695 PMC3888062

[212] Raja J Ludwig JM Gettinger SN Schalper KA Kim HS. Oncolytic virus immunotherapy: future prospects for oncology. J Immunother Cancer. (2018) 6:140. 10.1186/s40425-018-0458-z 30514385 PMC6280382

[213] Guo ZS Liu Z Kowalsky S Feist M Kalinski P Lu B. Oncolytic immunotherapy: conceptual evolution, current strategies, and future perspectives. Front Immunol. (2017) 8:555. 10.3389/fimmu.2017.00555 28555136 PMC5430078

[214] Woller N Gurlevik E Fleischmann-Mundt B Schumacher A Knocke S Kloos AM. Viral infection of tumors overcomes resistance to PD-1-immunotherapy by broadening neoantigenome-directed T-cell responses. Mol Ther. (2015) 23:1630–40. 10.1038/mt.2015.115 26112079 PMC4817928

[215] Gauvrit A Brandler S Sapede-Peroz C Boisgerault N Tangy F Gregoire M. Measles virus induces oncolysis of mesothelioma cells and allows dendritic cells to cross-prime tumor-specific CD8 response. Cancer Res. (2008) 68:4882–92. 10.1158/0008-5472.can-07-6265 18559536

[216] Errington F Steele L Prestwich R Harrington KJ Pandha HS Vidal L. Reovirus activates human dendritic cells to promote innate antitumor immunity. J Immunol. (2008) 180:6018–26. 10.4049/jimmunol.180.9.6018 18424722

[217] Boudreau JE Bridle BW Stephenson KB Jenkins KM Brunelliere J Bramson JL. Recombinant vesicular stomatitis virus transduction of dendritic cells enhances their ability to prime innate and adaptive antitumor immunity. Mol Ther. (2009) 17:1465–72. 10.1038/mt.2009.95 19401673 PMC2835245

[218] Rosenberg SA Restifo NP. Adoptive cell transfer as personalized immunotherapy for human cancer. Science. (2015) 348:62–8. 10.1126/science.aaa4967 25838374 PMC6295668

